# Iso-ADP-Ribose Fluorescence Polarization Probe for the Screening of RNF146 WWE Domain Inhibitors

**DOI:** 10.1021/acschembio.3c00512

**Published:** 2024-01-18

**Authors:** Kewen Peng, Ananya Anmangandla, Sadhan Jana, Yizhen Jin, Hening Lin

**Affiliations:** † Department of Chemistry and Chemical Biology, Cornell University, Ithaca, New York 14853, United States; ‡ Graduate Program of Biochemistry, Molecular and Cell Biology, Department of Molecular Biology and Genetics, Cornell University, Ithaca, New York 14853, United States; § Howard Hughes Medical Institute, Department of Chemistry and Chemical Biology, Department of Molecular Biology and Genetics, Cornell University, Ithaca, New York 14853, United States

## Abstract

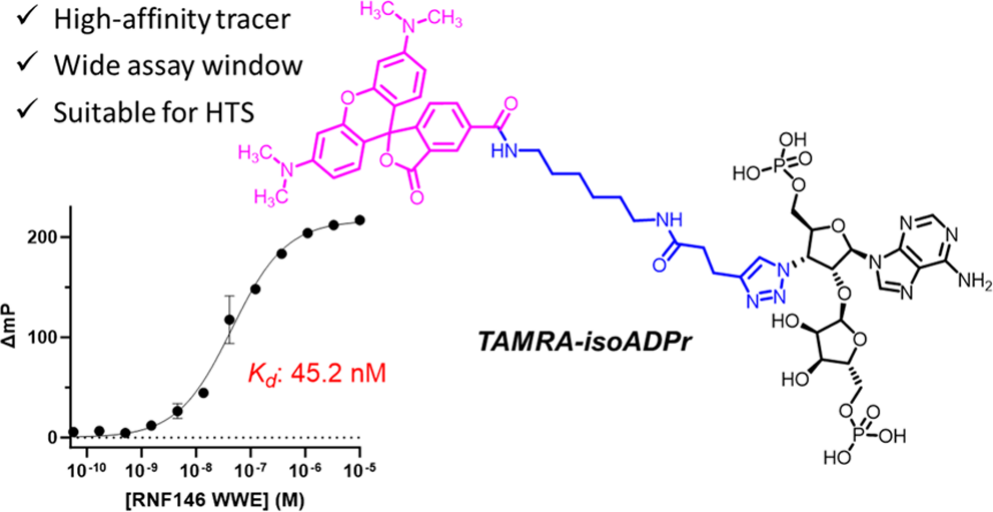

Poly-ADP-ribosylation is an important protein post-translational modification with diverse biological consequences. After binding poly-ADP-ribose on axis inhibition protein 1 (AXIN1) through its WWE domain, RING finger protein 146 (RNF146) can ubiquitinate AXIN1 and promote its proteasomal degradation and thus the oncogenic WNT signaling. Therefore, inhibiting the RNF146 WWE domain is a potential antitumor strategy. However, due to a lack of suitable screening methods, no inhibitors for this domain have been reported. Here, we developed a fluorescence polarization (FP)-based competition assay for the screening of RNF146 WWE inhibitors. This assay relies on a fluorescently tagged iso-ADP-ribose tracer compound, **TAMRA-isoADPr**. We report the design and synthesis of this tracer compound and show that it is a high-affinity tracer for the RNF146 WWE domain. This provides a convenient assay and will facilitate the development of small-molecule inhibitors for the RNF146 WWE domain.

## Introduction

As a post-translational modification (PTM), protein poly-ADP-ribosylation (PARylation) plays important roles in multiple biological processes. Using β-nicotinamide adenine dinucleotide (NAD^+^) as the substrate, poly-ADP-ribose polymerases (PARPs) catalyze the initiation, elongation, and branching of PARylation on target proteins. Subsequent recognition of these highly negatively charged poly-ADP-ribose (PAR) tags by PAR-binding modules-containing proteins (sometimes referred to as readers of PAR) triggers diverse biological effects.^1−4^

WWE domains, named after their most conserved tryptophan-tryptophan-glutamate residues, are an important class of PAR-binding modules present in many E3 ubiquitin ligases and several PARPs.^5^ Some WWE domains are reported to bind iso-ADP-ribose (isoADPr), the minimal internal unit of PAR, rather than the terminal or free ADP-ribose (ADPr), albeit WWE domains of different proteins may differ in their binding preferences and affinities.^6,7^ WWE domains of E3 ubiquitin ligases are of particular interest because of their role as an important link between protein PARylation and ubiquitination.^8^

Among WWE-containing E3 ubiquitin ligases, RING finger protein 146 (RNF146, also known as iduna) is well known for its critical role in PAR-dependent ubiquitination. Specifically, it has been shown that RNF146 can ubiquitinate axis inhibition protein 1 (AXIN1) after it is PARylated by tankyrase 1 (TANK1 or PARP5a), targeting AXIN1 for proteasomal degradation. As a consequence, the WNT signaling pathway is activated to promote cell survival, proliferation, and differentiation.^9^ Since the WNT signaling is abnormally activated in multiple cancers, several TANK1 inhibitors that can stabilize AXIN1 and attenuate the WNT signaling have been developed for cancer treatment in the past two decades.^10,11^ However, the safety profile of TANK1 inhibitors is still a question due to the observed on-target side effects, including intestinal toxicity^12^ and bone loss,^13^ and no TANK1 inhibitor has been approved so far. Therefore, strategies alternative to TANK1 inhibition are desired. To this end, RNF146 inhibition is promising in that it is expected to have similar effects on AXIN1 stability compared with TANK1 inhibition, given RNF146’s role in AXIN1 ubiquitination. At the same time, RNF146 inhibitors can potentially evade the side effects caused by direct TANK1 inhibitors.

RNF146 can be allosterically activated by PAR binding, and the structural explanation has been reported.^14^ TANK1 and RNF146 form a complex in which the RING domain of RNF146 is initially inactive, and TANK1 is responsible for substrate selection and PARylation. After TANK1-mediated protein PARylation, the internal isoADPr unit within the substrate PAR is recognized by the WWE domain of RNF146, triggering allosteric activation of RING E3, which ubiquitinates the substrate protein. Moreover, a single isoADPr molecule is enough to trigger the activation of RNF146 by interacting with the basic Lys61 residue in the RING domain. Small-molecule binders of the RNF146 WWE domain could mitigate PAR binding, thus inhibiting RNF146-mediated ubiquitination.

However, to date, there is not a single small-molecule inhibitor reported for the WWE domain of RNF146 or any other WWE domains. This is largely due to the lack of biochemical assays for detecting WWE domain binders. Here, we report the first binding assay for detecting or screening of RNF146 WWE inhibitors. We designed and synthesized a fluorescence polarization (FP) probe, **TAMRA-isoADPr**, and showed that it is a high-affinity binder for RNF146 WWE. The FP-based binding assay was then validated with a known binder, isoADPr, which yielded an IC_50_ value very similar to the reported *K*_d_ value. Finally, we showed that this assay can be used in a high-throughput manner with a satisfactory performance.

## Results and Discussion

A high-affinity tracer molecule is critical to an FP-based assay for the screening of RNF146 WWE inhibitor screening. Although a very recent paper^15^ used fluorescently labeled PARs as high-affinity tracers for PARP13 and RNF146 WWEs, a laborious purification process is required to obtain PAR with specific chain lengths. Moreover, the low yield of enzymatically synthesized PAR would restrict its use in inhibitor screening, where large quantities of tracer are often needed. Therefore, we aimed to obtain chemically tractable small-molecule tracers for RNF146 WWE, which are suitable for large-scale inhibitor screening.

**Figure 1:**
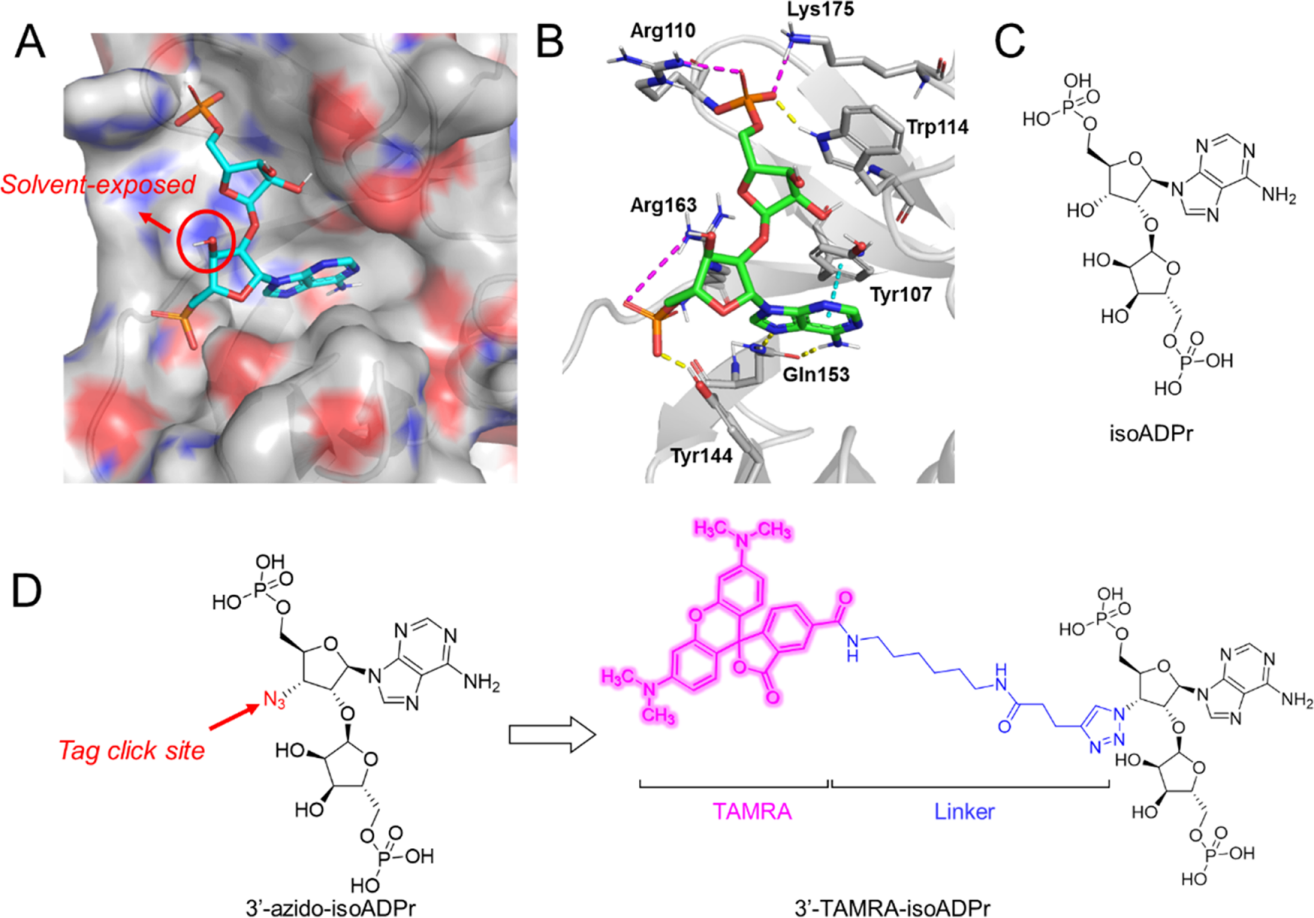
Design of the fluorescent polarization tracer, **TAMRA-isoADPr**. (A) Crystal structure of RNF146 WWE in complex with isoADPr (PDB ID: 3V3L). RNF146 WWE is shown in surface representation, and isoADPr is shown as sticks. Solvent-exposed 3′–OH of isoADPr is highlighted. (B) Interactions between isoADPr and RNF146 WWE. Hydrogen bonding, pi–pi stacking, and charge interactions are shown in yellow, cyan, and magenta dashes, respectively. (C) Chemical structure of isoADPr. (D) Design of **TAMRA-isoADPr** from 3-azido-isoADPr.

Since isoADPr (Figure 1C) is the only reported small-molecule ligand for RNF146 WWE, we first attempted to derivatize isoADPr based on the reported cocrystal structure of the isoADPr-RNF146 WWE complex. IsoADPr binds to RNF146 WWE in a “triangular” fashion (Figure 1A,B), with its two distal phosphate groups establishing extensive charged interactions with multiple arginine and lysine residues, including Arg110, Lys175, and Arg163, while its adenine ring is inserted into a binding cleft deeper inside the pocket by stacking with Tyr107 and interacting with Gln153.^6^ Since the proximal 3′–OH of isoADPr does not make any direct interaction with the protein and is solvent-exposed, we reasoned that this site might be an appropriate anchor point for the attachment of a fluorescent tag. Based on our previous success in designing FP tracer molecules for macrodomains,^16^ we decided to attach a tetramethylrhodamine (TAMRA) fluorophore at the 3′ position of isoADPr through a (6-aminohexyl)-3-(triazol-4-yl)propenamide linker (Figure 1D).

**Scheme 1:**
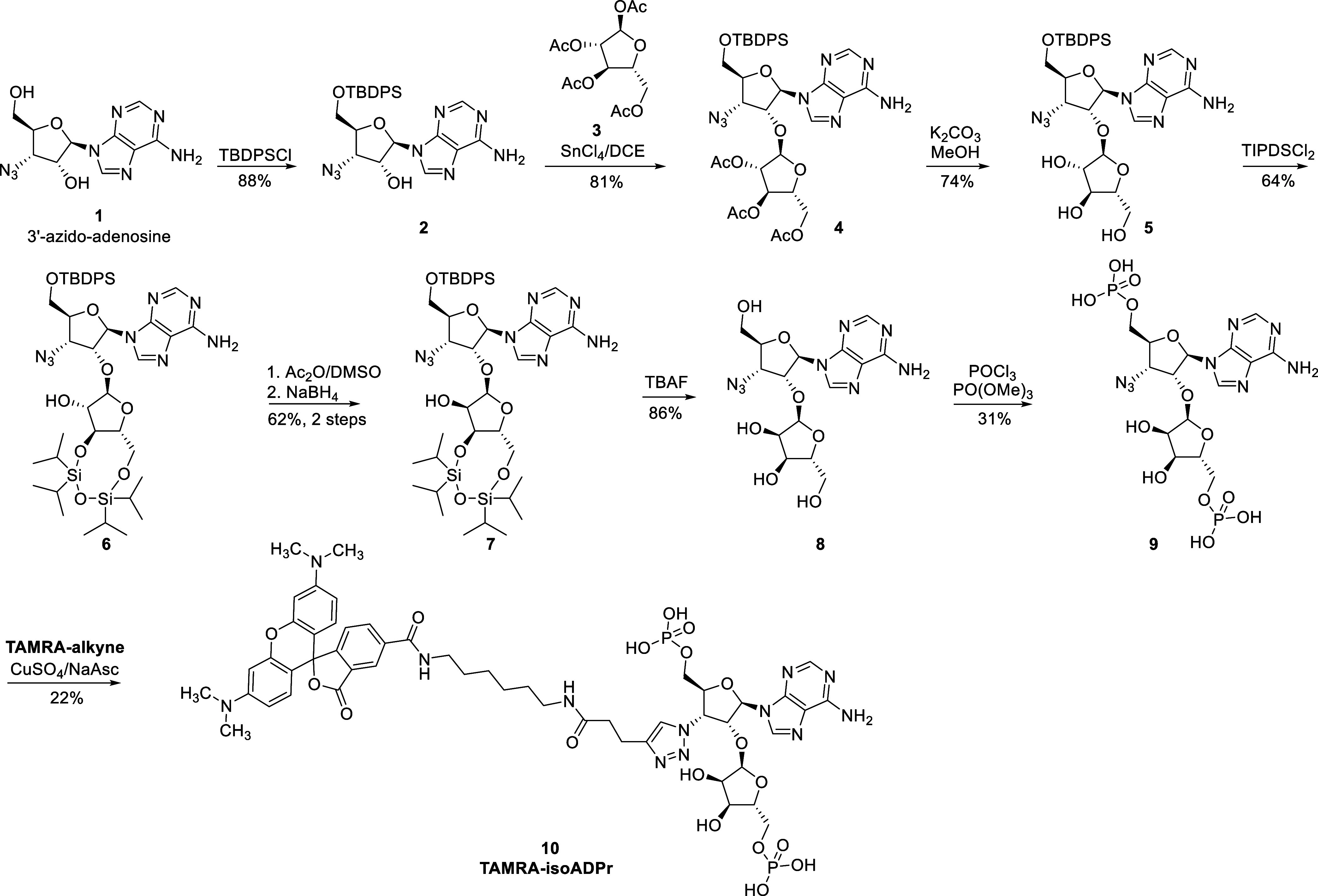
Synthetic Route for **TAMRA-isoADPr**

To synthesize the desired **TAMRA-isoADPr** (Scheme 1), we started with 3-azido-adenosine (**1**), which was prepared in multiple steps starting from α-D-xylofuranose through reported procedures.^17,18^ The 5′–OH of **1** was first protected with the *tert*-butyldiphenylsilyl (TBDPS) group selectively to give intermediate **2**. The critical α-1,2 glycosidic bond in disaccharide **4** was established through a SnCl_4_-activated O-glycosylation reaction between **2** and per-acetylated D-arabinofuranose **3**. This O-glycosylation reaction was stereospecific due to the participation of the neighboring 2-OAc. Deprotection of acetyl groups was achieved using potassium carbonate in methanol to afford intermediate **5**. Configurational inversion of the 2″–OH in **5** entails another four steps. First, the 3″,5″-diol was simultaneously protected with the 1,1,3,3-tetraisopropyldisiloxanylidene (TIPDS), followed by the epimerization of 2″–OH through a one-pot Robins oxidation–reduction sequence^19^ using Ac_2_O/DMSO as a mild oxidant and then NaBH_4_ as the reducing agent. Subsequently, all silyl protection groups were cleaved using tetra-*n*-butylammonium fluoride (TBAF) to afford the enantiomerically pure 2′-O-α-D-ribofuranosyl-3′-azidoadenosine (**8**). By carefully controlling the reaction time and temperature, selective and simultaneous phosphorylation of the two primary 5′- and 5″–OH groups of **8** was achieved with POCl_3_ in PO(OMe)_3_, affording the key intermediate 3′-azido-isoADPr (**9**) in moderate yield. It should be noted that significant amounts of 3′-azido-AMP and monophosphorylated byproducts were also formed, and normal-phase purification in the water–ammonia–isopropanol system was conducted to achieve good separation. Finally, click reaction of **9** with the previously reported **TAMRA-alkyne**(16) furnished the target molecule **TAMRA-isoADPr** (**10**).

**Figure 2:**
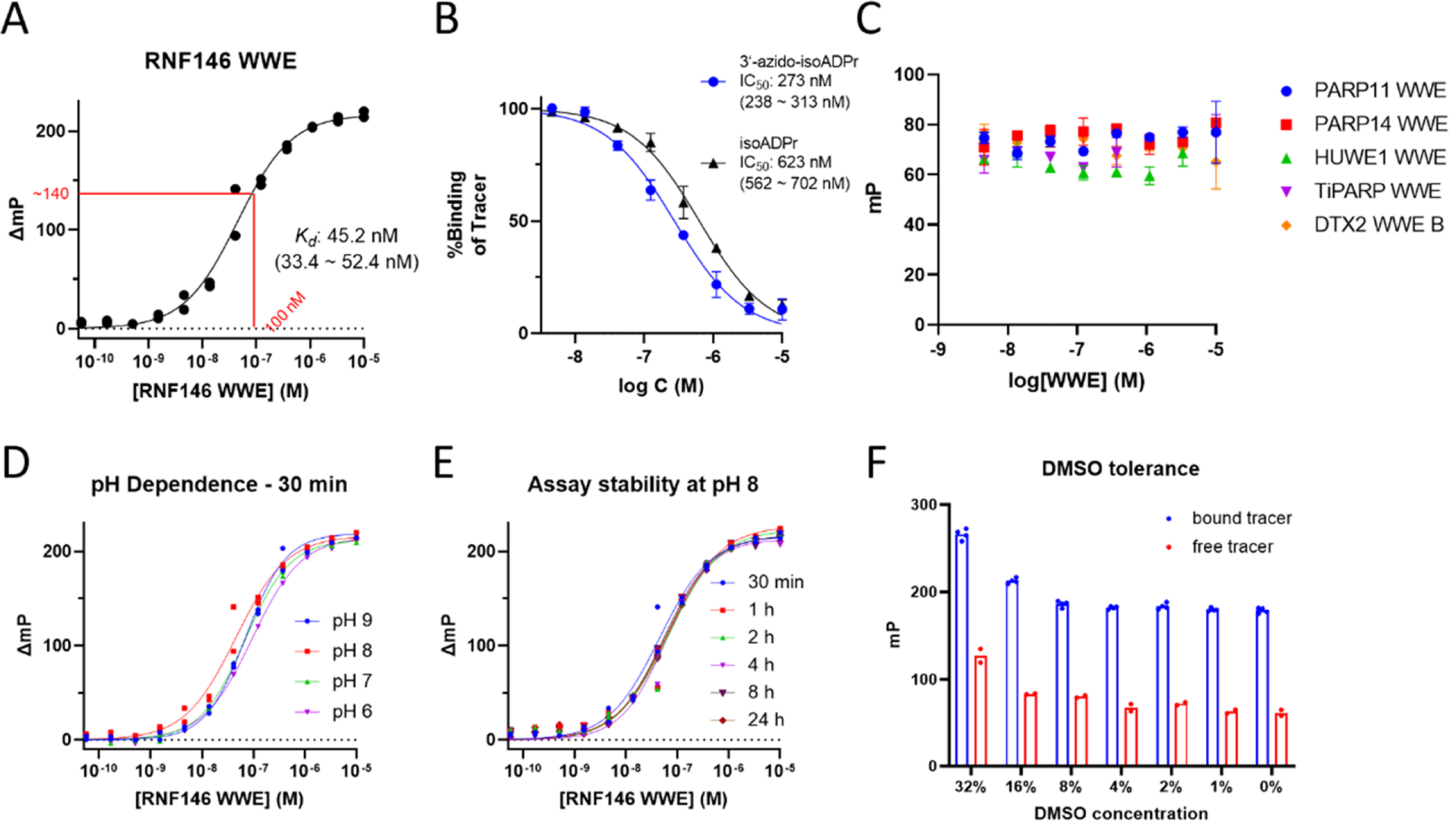
Data from fluorescence polarization assays. (A) Binding curve of **TAMRA-isoADPr** determined from RNF146 WWE titration. (B) IC_50_ curve of isoADPr and 3′-azido-isoADPr against RNF146 WWE domain determined in a competitive FP assay using **TAMRA-isoADPr** as the tracer. (C) Data for PARP11 WWE, PARP14 WWE, HUWE1 WWE, TiPARP WWE, and DTX WWE B showing no binding by **TAMRA-isoADPr**. (D) FP titration curves of **TAMRA-isoADPr** with RNF146 WWE in buffers with different pH values. (E) FP titration curves at pH 8 after different incubation time. (F) Effects of dimethyl sulfoxide (DMSO) concentration on the mP values of bound and free tracer. In panels (A–F), individual data points from each replicate are shown. In panels (B, C), plotted values are the mean ± SD (*n* = 2 or *n* = 3). For *K*_d_ and IC_50_, best-fit values are shown with the 95% confidence interval in parentheses.

Having obtained the tracer molecule **TAMRA-isoADPr**, we then examined its binding affinity by FP protein titration experiments, where a fixed concentration of the tracer (20 nM) was titrated with increasing concentrations of RNF146 WWE protein in Tris buffer (pH 8.0), yielding a binding curve with an increasing mP shift (ΔmP) until a plateau was observed. Gratifyingly, the resulting binding curve shows that **TAMRA-isoADPr** binds RNF146 WWE with high affinity (*K*_d_: 45.2 nM, Figure 2A), and only 100 nM RNF146 WWE protein is needed to achieve a favorable mP shift higher than 100.

**Figure 3:**
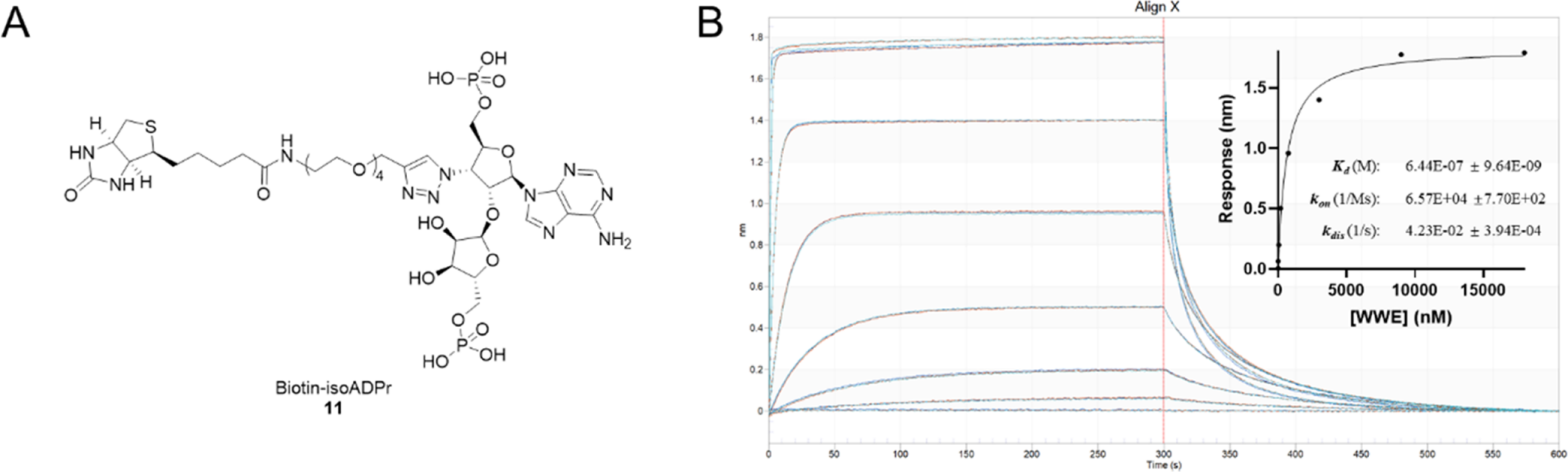
Biolayer interferometry experiment showing that immobilized **biotin-isoADPr** binds RNF146 WWE protein. (A) Chemical structure of **biotin-isoADPr**. (B) Processed BLI data showing response-time plot with fitted *K*_d_, *k*_on_, and *k*_off_ values represented as mean ± error calculated with the Octet BLI Analysis software (*n* = 3). The experiment was conducted using RNF146 WWE at 12, 49, 187, 750, 3000, 9000, and 18000 nM through 300 s association–dissociation cycles.

To further validate that our probe indeed can bind to RNF146 WWE, we sought to measure the *K*_d_ value of TAMRA-isoADPr toward RNF146 WWE through biolayer interferometry (BLI). A typical BLI experiment relies on an antibody-coated biosensor tip that can capture the protein of interest, and it was then dipped into solutions containing potential interacting molecules (here, referred to as the analyte).^20^ The interaction between the protein and the analyte can be measured in real time through monitoring the reflected light pattern upon complex formation on the biosensor surface, giving useful kinetics information, including *k*_on_, *k*_off_, and *K*_d_. Since RNF146 WWE was expressed with an N-terminal His tag, we first tried to immobilize it onto an anti-His biosensor (HIS1K), but we observed a significant decrease in the baseline signal after protein loading (Figure S1A), suggesting the affinity between the protein and the antibody coated on the biosensor was low and the immobilized protein was washed off easily. Indeed, the streptavidin biosensors (SA and SSA) are more commonly used in the literature^20−22^ and usually provide more stable baselines and more sensitive measurements. Therefore, we synthesized a biotin-labeled isoADPr compound (**11**, Figure 3A) from 3′-azido-isoADPr, which is structurally analogous to **TAMRA-isoADPr**. **Biotin-isoADPr** was immobilized onto SA biosensors that were then dipped into RNF146 WWE solutions of varying concentrations in multiple association–dissociation cycles (Figure S1B). The results indicate that immobilized **biotin-isoADPr** binds RNF146 WWE with high on- and off-rates, typical of protein–small molecule binding (Figure 3B). Importantly, the fitted *K*_d_ value of this interaction is 644 nM, which is comparable to the reported *K*_d_ value (370 nM) of isoADPr measured through isothermal titration calorimetry experiments.^6^ The BLI experiment further supports that our tracer design strategy works for RNF146 WWE.

We then screened the tracer TAMRA-isoADPr against WWE domains of some other proteins, including PARP11, PARP14, HUWE1, TiPARP, and DTX2, under the same conditions. However, none of these WWE domains could bind the tracer (Figure 2C). To check the folding of purified WWE domains, we obtained the circular dichroism (CD) spectra for the purified WWE domains, and the results indicated proper folding of all purified proteins (Figure S5). Although disappointing, this result was not entirely unexpected because the tracer was designed based on the structure of isoADPr binding to RNF146 WWE. The isoADPr binding pocket of RNF146 WWE is special in that the entire pocket is largely positively charged with multiple arginine and lysine residues to interact with both phosphate groups of isoADPr, while this feature is missing in other WWE domains containing proteins (Figure S2). Indeed, RNF146 WWE is the only reported high-affinity binder of the isoADPr monomer, so it is likely that other WWE domains tested are simply too weak binders to show any significant binding at the concentrations tested. For instance, HUWE1 WWE was reported to bind isoADPr with a *K*_d_ value of 13 μM,^6^ and it may bind ADPr instead.^23^ However, our previously developed **TAMRA-ADPr** does not bind the WWE domains tested either (Figure S3), suggesting the TAMRA tag placed at the distal ribose ring of ADPr may not be tolerated by HUWE1 WWE or it is possible HUWE1 recognizes ADP-ribosylated proteins through multivalency rather than tight binding.

We next tried to optimize the assay conditions. We first varied the pH from 6 to 9 (Figure 2D). While pH 8 gave the strongest binding, the effect of pH was relatively small. Different incubation time ranging from 30 min to 24 h also did not affect the result much (Figure 2E), suggesting that tracer binding reached equilibrium within 30 min and the assay was stable up to 24 h. Thus, the incubation time was set to be 30 min in later experiments. DMSO tolerance of this assay was then examined for potential drug screening purposes (Figure 2E). Using 100 nM RNF146 WWE, the mP values of bound and unbound tracer remained consistent up to 16% DMSO. While a significant increase in the mP values was observed in 32% DMSO, the ΔmP did not decrease at this DMSO concentration. Therefore, this assay is suitable for screening applications that use less than 16% DMSO.

Having optimized the assay conditions, we then sought to establish a competitive FP-based binding assay for the screening of potential RNF146 WWE inhibitors, where an inhibitor candidate and **TAMRA-isoADPr** were incubated with RNF146 WWE. If the candidate compound could compete with the tracer for protein binding, then a lowered mP shift value would be expected. Since **TAMRA-isoADPr** is a strong binder for the RNF146 WWE domain, low concentrations of the protein (50–100 nM) could achieve a satisfactory assay window of 100 mP, rendering this assay cheap and suitable for high-throughput screening (HTS) campaigns. We first validated this competitive binding assay with isoADPr, a known ligand for RNF146 WWE, using **TAMRA-isoADPr** at 20 nM, RNF146 WWE protein at 100 nM, and isoADPr at increasing concentrations in 96-well plates. The IC_50_ of isoADPr (Figure 3B) was calculated from the FP binding curve to be 623 nM, which is comparable to the reported *K*_d_ value of 370 nM. In our previous study on FP assay for macrodomains,^16^ we found the **ADPr-N**_**3**_ precursor is more potent than ADPr for SARS-CoV-2 Macro1. Here, we also tested the 3′-azido-isoADPr precursor and found it is 2-fold more potent than isoADPr for RNF146 WWE. This result can be explained by the lower desolvation costs of azido groups compared with hydroxyl groups. Indeed, the removal of nonessential hydroxyl groups is a commonly used strategy in glycomimetic drug design.^24^ Overall, our competitive FP binding assay is suitable for measuring the binding affinity of potential RNF146 WWE inhibitors.

**Figure 4:**
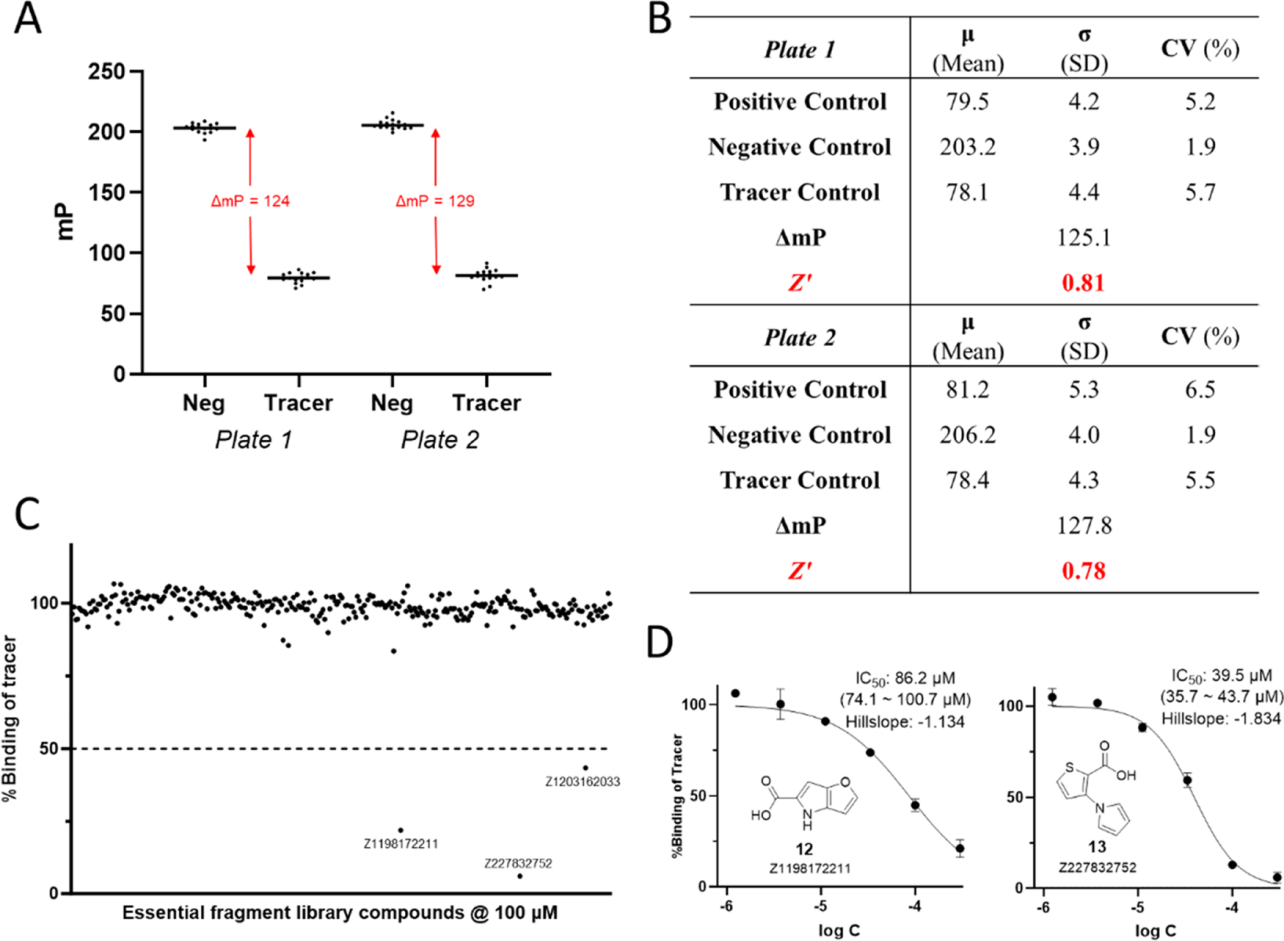
Pilot screen of the Enamine Essential Fragment Library using the developed assay. (A) Negative control and positive control data from two replicate plates. The assay window (ΔmP) was calculated as the difference between mean mP values of negative and tracer control wells. (B) Summary of some important screen parameters, including ΔmP and *Z*’ calculated from the mP values of control wells. (C) Data from fragment screening. Compound IDs for the three hits that displaced >50% tracer binding are shown. (D) IC_50_ curves and chemical structures of two fragment hits. Plotted values are the mean ± SD (*n* = 3). For IC_50_, best-fit values are shown with the 95% confidence interval in parentheses.

To test the performance of the established FP assay in high-throughput formats, we conducted a pilot screen where isoADPr was used as the positive control, and Enamine Essential Fragments Library, which contains 320 fragments, was used as a test case for potential inhibitors. The test library was screened at 100 μM in 1% DMSO under the established conditions in duplicates in two 384-well plates (Figure 4A). In each plate, four controls were used: buffer control, tracer control, negative control, and positive control. Buffer control allowed the detection of background fluorescence, while tracer and negative controls gave mP values of the free tracer and maximally bound tracer, to which inhibition rates of fragment candidates were normalized. Positive control wells contained 100 μM isoADPr, which should completely displace the tracer from target binding and hence have an inhibition rate of ∼100%. From these control wells, different assay parameters could be calculated. One important FP assay parameter is the assay window (ΔmP), which is calculated as the difference between the mP value recorded for the bound tracer (i.e., negative control) and the mP value recorded for the free tracer (i.e., tracer control). A minimum assay window of 70 mP has been suggested to achieve satisfactory performance.^25^ Another parameter general to all HTS assays is the screening window coefficient (*Z*′ factor). A *Z*′ factor value between 0.5 and 1 is suitable for HTS.^26^ In the pilot experiment, both plates demonstrated promising assay performance with *Z*′ factor values of 0.81 and 0.78, and ΔmPs of 125 and 128, respectively (Figure 4B). Moreover, the coefficients of variation (CV) of both negative and positive control wells were low.

Since compounds can have intrinsic fluorescence at tested wavelengths, we first checked the intrinsic fluorescence intensities of all library compounds at the tested concentration of 100 μM in the assay buffer. After eliminating eight compounds with high fluorescence readings (three times higher than buffer; see the Supporting Information), we identified three compounds that demonstrated inhibition rates higher than 50% (Figure 4C) in both plates. After confirming the hits’ identities by liquid chromatography-mass spectrometry (LC-MS), we measured their IC_50_ values using the established FP assay. Gratifyingly, we were able to obtain well-behaved IC_50_ curves of two hit compounds (**12**, Z1198172211 and **13**, Z104476320, Figure 4D) with estimated IC_50_ values of 86 and 40 μM, respectively, while another hit, Z1203162033, turned out to be a much weaker binder (Figure S4). Thus, our pilot screen readily detected the positive control isoADPr and identified two fragment hits from a commercial fragment library. We believe our assay is well suited for the future screening of more sophisticated libraries that will yield more lead-like hits and facilitate the development of RNF146 WWE inhibitors.

## Conclusions

In summary, we developed an FP-based competition assay for the screening of RNF146 WWE inhibitors through the synthesis of **TAMRA-isoADPr** as the tracer. We demonstrated that this assay can accurately capture ligand binding affinity and is suitable for HTS. We believe this assay will be useful in identifying RNF146 WWE inhibitors and validating RNF146 WWE as a druggable target. Of the tested domains, this assay only works for RNF146 WWE, likely because other WWE domains do not bind isoADPr very strongly. Future endeavors to design probe molecules that can bind WWE domains of other proteins are warranted for inhibitor discovery targeting these proteins, which may help shed light on their biological functions and druggability. Additionally, we devised a feasible synthetic route to 3′-azido-isoADPr, which we believe will be useful for the future synthesis of isoADPr-based probes or prodrugs targeting RNF146 WWE or other PAR-binding proteins.

## Materials and Methods

### Reagents

isoADPr was synthesized and purified as previously described.^6^ The Enamine Fragment Library (Catalog Number: ESS-320–100-X-100) was purchased from Enamine. Unless otherwise noted, all biological reagents and consumables were purchased from commercial vendors.

### Chemical Synthesis

Detailed synthetic procedures can be found in the Supporting Information.

### Expression and Purification of WWE Domains

The HUWE1 WWE domain was purified as previously reported.^23^ PARP11, PARP14, TiPARP, DTX2, and RNF146 WWE domain plasmids were either cloned or purchased from Twist Biosciences using NdeI/XhoI cut sites in pET28a vectors (full sequences available in the SI). The plasmids were transformed into BL21(DE3) chemically competent *Escherichia coli*. Four L portion of LB broth with 50 μg/mL kanamycin was inoculated with an overnight starter grown at 37 °C. Cultures were grown at 200 rpm and 37 °C for ∼4 h until the OD600 reached 0.8. Then, IPTG was added to 0.5 mM, and the cells were incubated at 16 °C overnight to allow protein expression. Cells were harvested by centrifugation for 6000*g*. Cell pellets were frozen at −80 °C or immediately used for purification. Pellets were resuspended in lysis buffer (50 mM Tris (pH 8.0) and 500 mM NaCl, 0.5 mg mL^–1^ lysozyme, 1 mM PMSF, and Pierce universal nuclease). Following a 30 min incubation, cells were sonicated on ice for 4 min in total at 60% amplitude. Lysate was clarified at 4 °C and 30,000 g for 35 min. Clarified lysate was loaded onto Ni-NTA resin, washed with 50 mL of wash buffer (50 mM Tris pH 8.0, 500 mM NaCl, 20 mM imidazole), and eluted with elution buffer (50 mM Tris pH 8, 500 mM NaCl, 200 mM imidazole). Crude WWE domains were concentrated using a 10-kDa MWCO Amicon filter and loaded onto a HiLoad 16/600 Superdex 75 gel filtration column equilibrated with storage buffer (25 mM Tris at pH 8.0, 150 mM NaCl, 10% glycerol) on a KTA FPLC system. Fractions containing WWE domains were pooled, concentrated, flash-frozen in liquid nitrogen, and stored at −80 °C for future use.

### Biolayer Interferometry

The binding of **biotin-isoADPr** to the RNF146 WWE was monitored and measured on an Octet RH16 biolayer interferometer. Three replicate streptavidin biosensor tips (SA) were loaded with 1 μM **biotin-isoADPr** in the kinetics buffer (PBS with 0.02% Tween-20 and 0.1% BSA) for 300 s. After a 300 s baseline step, the loaded sensor tips were moved to sample wells containing RNF146 WWE protein at 0, 12, 49, 187, 750, 3000, 9000, and 18000 nM in the kinetics buffer sequentially in multiple cycles, with each cycle consisting a 300 s association step in the sample well and a 300 s dissociation step in the buffer well. The volume of each well was 200 μL. A reference biosensor without **biotin-isoADPr** loading was used to exclude the possibilities of nonspecific binding, and reference wells without RNF146 WWE protein were used for blank subtraction. Data were processed and curves were fitted with a 1:1 best-fit model in Octet BLI Analysis software.

### FP Titration of WWE Domains

The purified WWE domain proteins were 3-fold serially diluted from 20 μM to ∼5 nM in the assay buffer (25 mM Tris pH 8.0, 150 mM NaCl, and 0.01% Tween-20). In the assay, 50 μL of the protein solution at each concentration was transferred to a 96-well black plate (Corning, #3915), followed by the addition of 50 μL of **TAMRA-isoADPr** (40 nM, 2X) in the assay buffer to reach a final volume of 100 μL. The plate was allowed to stand at RT for 30 min and then scanned on Cytation5 equipped with an FP filter cube (Agilent, part number: 8040562, Ex: 530/25, Em: 590/35). mP values were calculated using the equation below
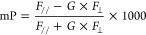
where *F*_//_ and *F*_⊥_ are the parallel and perpendicular fluorescence intensities, respectively, and *G* is the grating factor of the instrument, which was calibrated so that 20 nM 5-TAMRA has an mP shift of 50. The obtained mP data were fitted in the one-site-specific binding model implemented in GraphPad Prism 9.4.1 (GraphPad Software, Inc.) to give the *K*_d_ value using the equation below
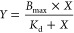


### FP-Based Binding Assay for RNF146 WWE

The procedure is adapted from our previous work.^16^ Briefly, the RNF146 WWE protein (200 nM, 2X) was mixed with **TAMRA-isoADPr** (40 nM, 2X) in the assay buffer to give the protein–tracer mixture. To each well of a 96-well black plate was added 50 μL protein–tracer mixture solution, followed by the addition of 50 μL of the compound solution (2X final concentration) in the assay buffer to reach a final volume of 100 μL. The plate was allowed to stand at RT for 30 min and was then scanned on Cytation5 equipped with an FP filter cube (Agilent, part number: 8040562, Ex: 530/25, Em: 590/35). The relative percent binding of the tracer was calculated as follows
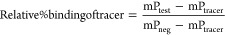
where mP_test_, mP_tracer_, and mP_neg_ are mP values of the test wells, tracer control wells, and negative control wells, respectively. The obtained data were then fitted into an IC_50_ curve using the sigmoidal four-parameter logistic model implemented in GraphPad Prism 9.4.1 (GraphPad Software, Inc.) using the equation below
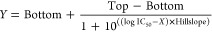
where *Y* is the relative percent binding of the tracer, and Top and Bottom were constrained to be 100 and 0, respectively.

### Fragment Library Screening

Using epMotion 96 (Eppendorf), 49.5 μL of the assay solution (100 nM RNF146 WWE and 20 nM **TAMRA-isoADPr**) or control solution (tracer-only, buffer-only, and positive control with additional 100 μM isoADPr) was transferred from a 96-well mother plate to a 384-well black plate (Corning, #3575). Next, 0.5 μL of 10 mM fragment in DMSO was added into each well, and 0.5 μL DMSO was added into all control wells. The plate was allowed to stand at RT for 30 min before being scanned, as described above. The screening was conducted in duplicate. For each fragment and positive and negative control well, the relative percent binding of the tracer was calculated as described above. *Z*’ factor was calculated with the following equation
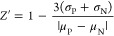
where σ_P_ and σ_N_ are the standard deviations of ΔmP values of positive and negative control wells, while μ_P_ and μ_N_ are the mean ΔmP values of positive and negative control wells.

## Supporting Information Available

Raw data of BLI experiments, ESP surface comparison of WWE domains, and FP titration of WWE domains with **TAMRA-ADPr** (Figures S1–S4); procedures of protein purification and chemical synthesis with characterization data (PDF)
Raw and processed data of fragment screening (XLSX)
