# Supplementary material for: Iso-ADP-Ribose Fluorescence Polarization Probe for the Screening of RNF146 WWE Domain Inhibitors
Source: ACS Chem Biol. 2024 Jan 18;19(2):300–7. doi: 10.1021/acschembio.3c00512 (PMC10877565; doi:10.1021/acschembio.3c00512)

Supporting Information

An isoADP-ribose fluorescence polarization probe for the screening of RNF146 WWE domain inhibitors

Kewen Peng[1], Ananya Anmangandla[1], Sadhan Jana[1], Yizhen Jin[2], Hening Lin[3,*]

[1]Department of Chemistry and Chemical Biology, Cornell University, Ithaca, NY 14853, USA

[2]Graduate Program of Biochemistry, Molecular and Cell Biology, Department of Molecular Biology and Genetics, Cornell University, Ithaca, New York 14853, USA

[3]Howard Hughes Medical Institute; Department of Chemistry and Chemical Biology; Department of Molecular Biology and Genetics, Cornell University, Ithaca, NY 14853, USA

*Correspondence: hl379@cornell.edu

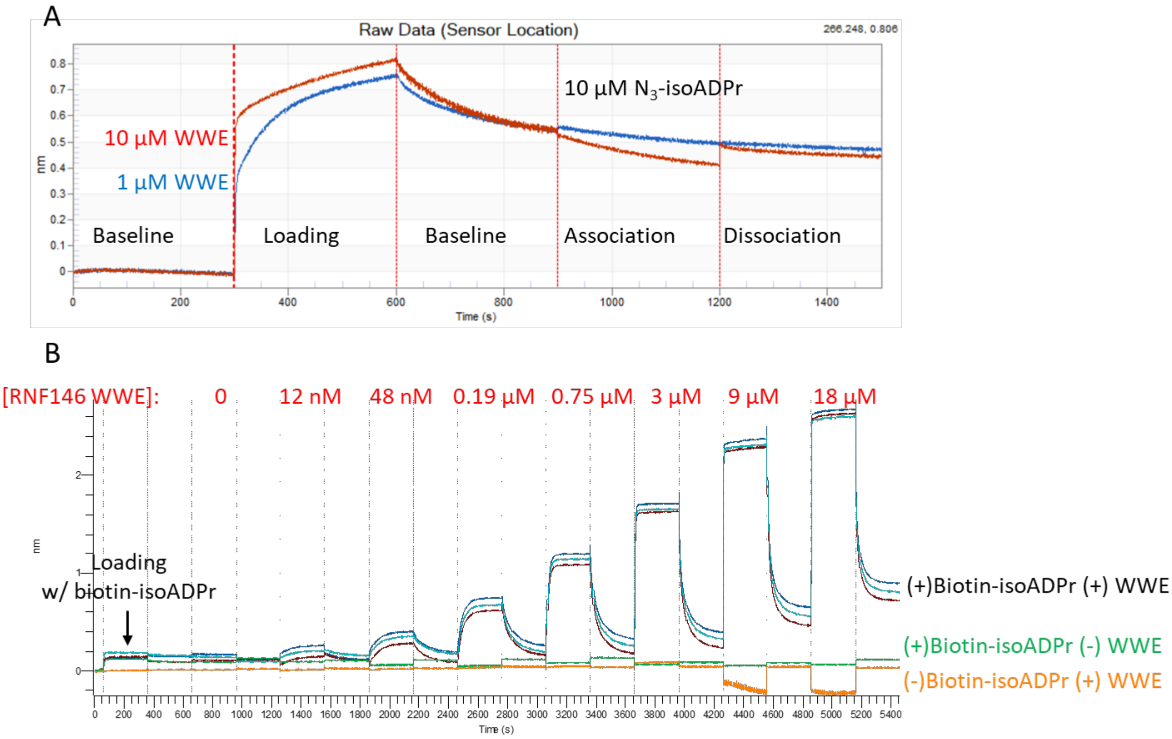

Figure S1. Raw data of biolayer interferometry experiments. (**A**) Attempts to load HIS1K biosensors with RNF146 WWE led to declining baselines. (**B**) Dose-dependent signal using SA biosensors loaded with biotin-isoADPr.

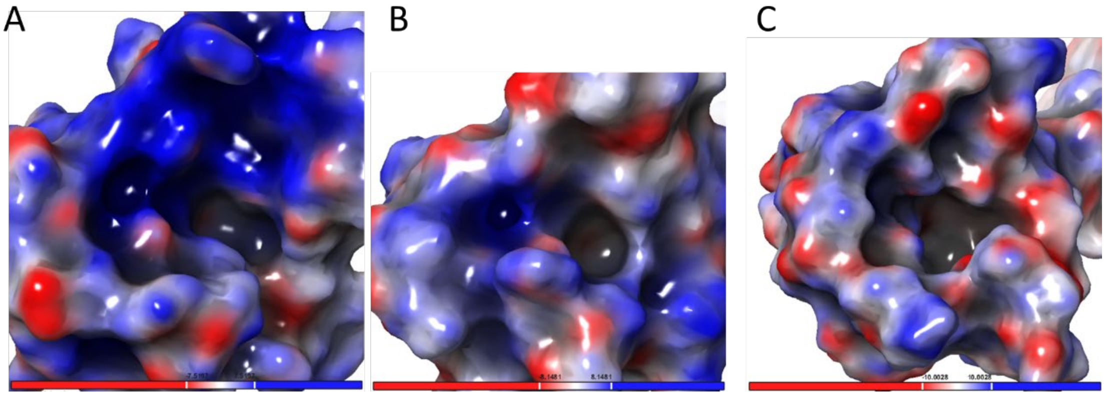

Figure S2. Electrostatic potential (ESP) surfaces of (**A**) RNF146 WWE (PDB ID: 3V3L) (**B**) HUWE1 WWE (PDB ID: 6PFL) and (**C**) PARP11 WWE (PDB ID: 2DK6). The view is centered on the isoADPr binding pocket of RNF146 WWE and the other two proteins were aligned to RNF146 WWE. The ESP surfaces were created with the Poisson-Boltzmann ESP program in Schrodinger.

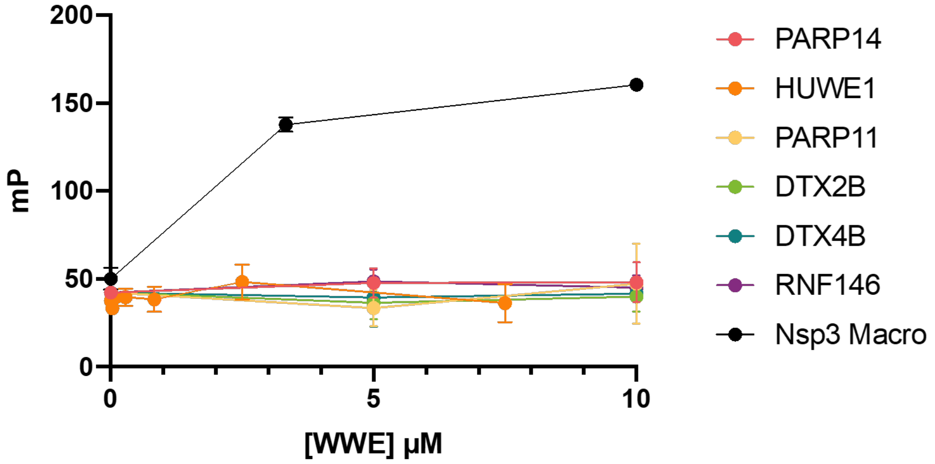

Figure S3. FP titration of different WWE domains using **TAMRA-ADPr** (n=3). Data of SARS-CoV2 Nsp3 Macro (black) is shown here as comparison.

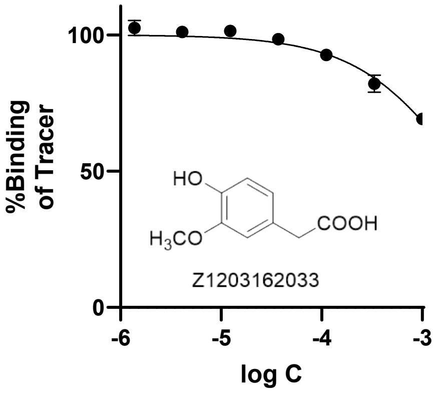

Figure S4. Attempted IC_{50} measurement and chemical structure of Z1203162033.

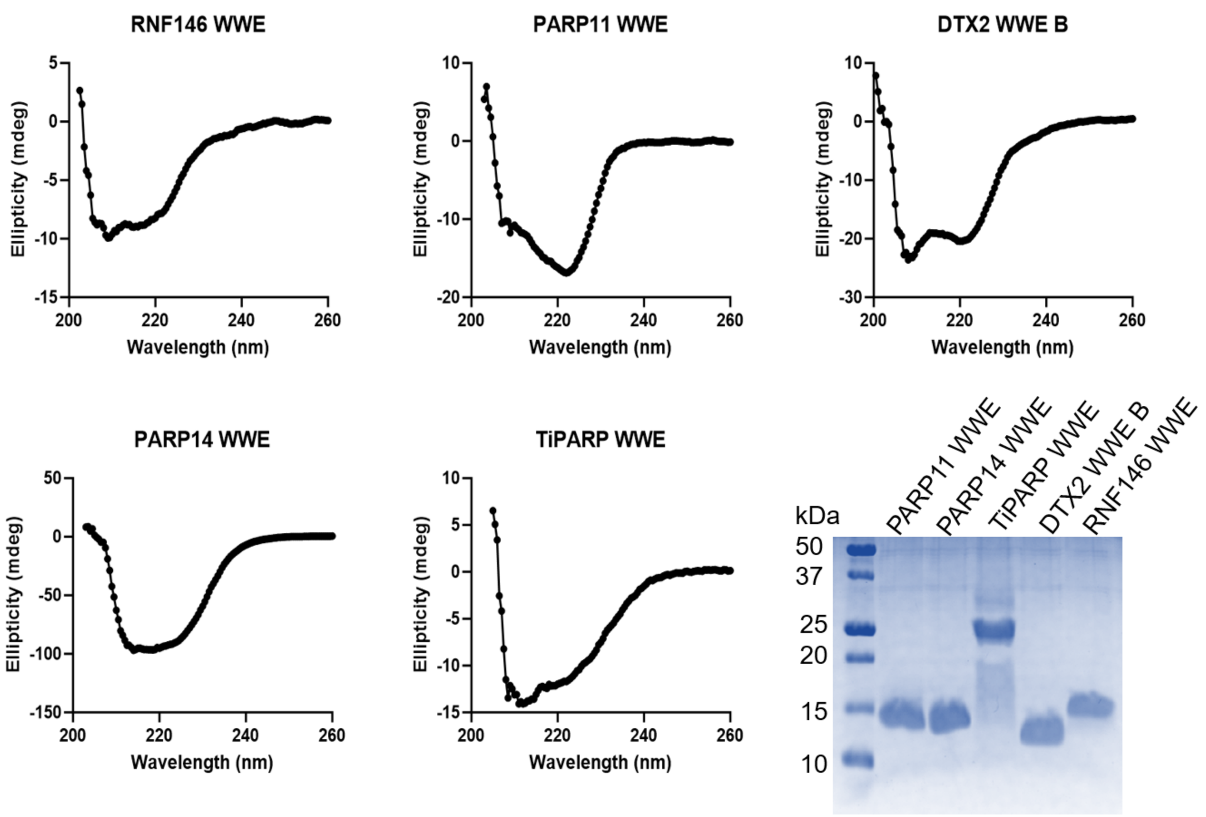

Figure S5. Circular dichroism measurements of purified WWE domains. The experiment was conducted with a JASCO J-1500 CD Spectrometer at 25℃ in Tris buffer pH 8.0. The protein gel showing all the purified WWE domain proteins is also shown.

Supplementary Methods

Design of WWE domain plasmids

PARP11, PARP14, TiPARP, RNF146 and DTX2 WWE domain plasmids were either cloned or purchased from Twist Biosciences and were designed as indicated below. PARP11, PARP14, TiPARP, RNF146 and DTX2 WWE domains were cloned into pET28a using NdeI and XhoI cut sites with a N-terminal His tag.

PARP11 WWE (aa31-110) (NM_020367.6)

```
DTQWGWFYLAECGKWHMFQPDTNSQCSVSSEDIEKSFKTNPCGSISFTTSKFSYKIDFAEMKQMNLTTGKQRLIK
RAPFS
GATACCCAGTGGGGCTGGTTTTACTTGGCAGAATGTGGGAAGTGGCACATGTTTCAGCCGGATACCAACAGTCAG
TGTTCAGTTAGCAGTGAAGATATCGAAAAAAGCTTCAAAACAAACCCTTGTGGCTCCATTTCTTTTACTACTTCC
AAATTCAGCTACAAGATAGACTTTGCAGAAATGAAGCAAATGAATCTCACCACTGGAAAGCAGCGCTTAATAAAA
AGAGCCCCCTTTTCT
```

PARP14 WWE (aa1522-1601) (NM_017554.3)

```
EQESRADCISEFIEWQYNDNNTSHCFNKMTNLKLEDARREKKKTVDVKINHRHYTVNLNTYTATDTKGHSLSVQR
LTKS
GAACAGGAATCCCGGGCAGATTGTATCAGTGAGTTTATAGAATGGCAGTATAATGACAATAACACTTCTCATTGT
TTTAACAAAATGACCAATCTGAAATTAGAGGATGCAAGGAGAGAAAAGAAAAAAACAGTTGATGTCAAAATTAAT
CATCGGCACTACACAGTGAACTTGAACACATACACTGCCACAGACACAAAGGGCCACAGTTTATCTGTTCAGCGC
CTCACGAAATCC
```

DTX2 WWE B (aa103-179) (NM_001102594.3)

```
HSAPGRGVVWEWLSDDGSWTAYEASVCDYLEQQVARGNQLVDLAPLGYNYTVNYTTHTQTNKTSSFCRSVRRQAG
PP
CACTCAGCCCCTGGCCGAGGTGTCGTCTGGGAGTGGCTGAGCGACGATGGCTCCTGGACTGCCTATGAAGCCAGC
GTCTGTGACTATCTGGAGCAGCAGGTGGCCAGGGGCAACCAGCTCGTGGACTTGGCCCCCCTGGGGTACAACTAC
ACTGTCAACTACACCACCCACACGCAGACCAACAAGACTTCCAGCTTCTGCCGCAGCGTGCGGCGCCAAGCAGGG
CCGCCT
```

RNF146 WWE (aa92-184) (NM_001242850.1)

```
MEELKAASRGNGEYAWYYEGRNGWWQYDERTSRELEDAFSKGKKNTEMLIAGFLYVADLENMVQYRRNEHGRRRK
IKRDIIDIPKKGVAGLRLD
GAAGAACTCAAGGCAGCAAGTAGAGGAAATGGTGAATATGCATGGTATTATGAAGGAAGAAATGGGTGGTGGCAG
TACGATGAGCGCACTAGTAGAGAGCTGGAAGATGCTTTTTCCAAAGGTAAAAAGAACACTGAAATGTTAATTGCT
GGCTTTCTGTATGTCGCTGATCTTGAAAACATGGTTCAATATAGGAGAAATGAACATGGACGTCGCAGGAAGATT
AAGCGAGATATAATA
```

TiPARP WWE (aa258-416) (NM_015508.5)

```
LKHHTVLPYHWQIKRTTTQKWQSVFNDSQEHLERFYCNPENDRMRMKYGGQEFWADLNAMNVYETTEFDQLRRLS
```

TPPSSNVNSIYHTVWKFFCRDHFGWREYPESVIRLIEEANSRGLKEVRFMMWNNHYILHNSFFRREIKRRPLFRS
CFILLPYLQ
TTGAAGCACCACACTGTCTTGCCATATCATTGGCAGATCAAAAGGACAACTACTCAAAAGTGGCAGAGTGTATTC
AATGATTCTCAGGAGCACTTGGAAAGATTTTACTGTAACCCAGAAAATGATAGAATGAGAATGAAGTATGGAGGA
CAAGAATTTTGGGCAGATTTGAATGCCATGAACGTGTATGAAACAACTGAATTTGACCAACTACGAAGGCTGTCC
ACACCACCCTCTAGCAATGTCAACTCTATTTACCACACAGTCTGGAAATTCTTCTGTAGGGACCACTTTGGATGG
AGAGAGTATCCCGAGTCTGTCATTCGATTGATTGAAGAAGCCAACTCTCGGGGTCTGAAAGAGGTTCGATTTATG
ATGTGGAATAACCACTACATCCTCCACAATTCATTCTTCAGGAGAGAGATAAAAAGGAGACCCCTCTTCCGCTCC
TGTTTTATACTGCTTCCATATTTACAG

General synthetic methods

Unless otherwise stated, all reactions were carried out under ambient atmosphere. All reagents and solvents were purchased from Sigma Aldrich, TCI, Alfa-aesar, Combi-blocks, Chem-impex international, Ambeed, VWR, and Fisher scientific and were used as received. Thin layer chromatography (TLC) was performed on precoated glass-backed silica gel 60 F254 plates (EMD Millipore), visualized by UV or staining with 10% H_2SO_4 in ethanol. SiliaFlash® P60 (SiliCycle) silica gel was used for column chromatography purification. All unreported compounds were characterized by ^{1}H-NMR, ^{13}C-NMR and/or ^{31}P-NMR spectra recorded on a 500MHz Bruker spectrometer. Mass spectra (MS) were obtained on a LCQ Fleet Mass Spectrometer (Thermo Scientific) equipped with an electrospray ionization (ESI) source connected to a Shimadzu HPLC LC20-AD. High-resolution mass spectra (HRMS) were acquired on an ESI ion source (IonSense) coupled to an Exactive Orbitrap mass spectrometer (Thermo Scientific). Preparative HPLC was run on a Hypersil C18 column (5 μm, 30 mm * 150 mm) with water (0.1% TFA) as solvent A and acetonitrile (0.1% TFA) as solvent B, following a linear gradient of solvent B from 0% to 50% in 45 minutes.

Synthesis of TAMRA-isoADPr

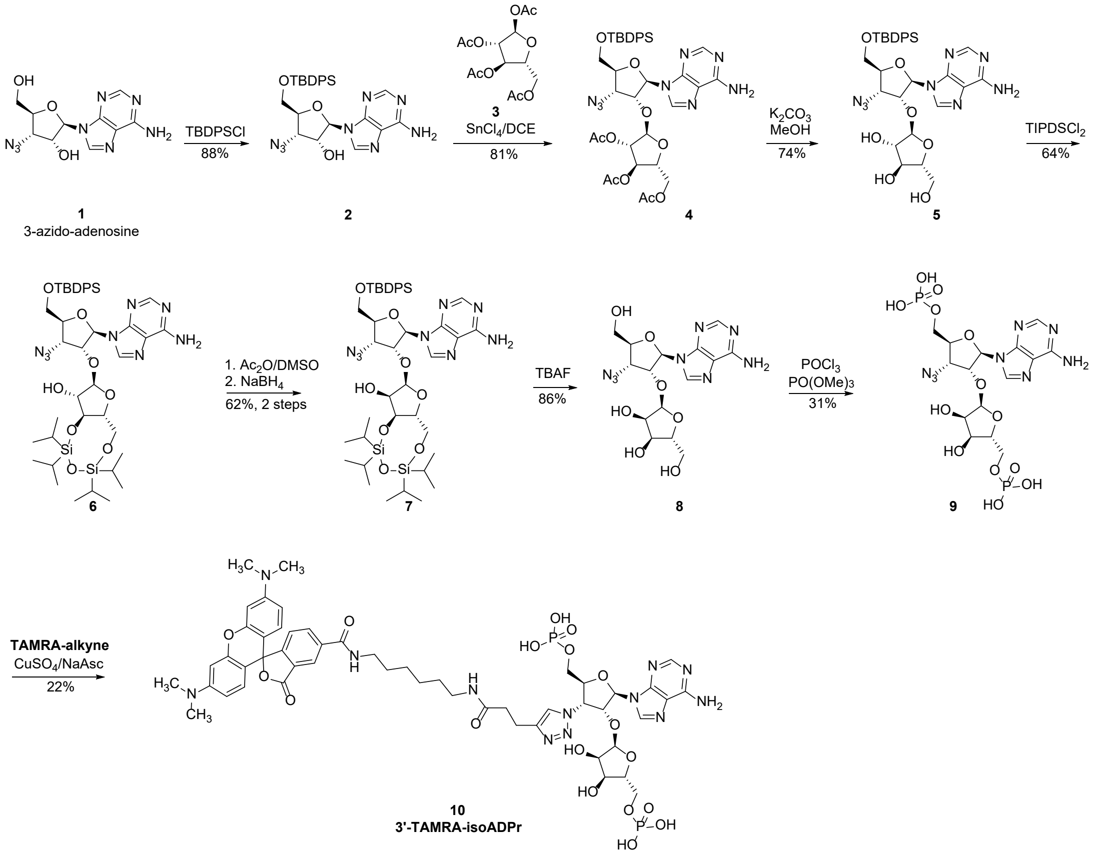

*9-(3-azido-3-deoxy-5-O-tert-butyldiphenylsilyl-β-D-ribofuranosyl)-adenine (**2**)*

To a stirring solution of **1** (260 mg, 0.89 mmol, 1 eq.) in dimethylformamide (DMF, 2 mL) was added imidazole (151 mg, 2.22 mmol, 2.5 eq.), followed by the addition of TBDPSCl (277 µL, 1.07 mmol, 1.2 eq.) in an ice bath. The reaction mixture was gradually warmed to room temperature and stirred overnight. Upon full conversion of the starting material, the reaction was quenched with water (6 mL) and the resulting mixture was extracted with ethyl acetate (10 mL, twice). The organic layer was combined and washed with brine before evaporation to dryness. The residue was subjected to silica column purification (dichloromethane or DCM:MeOH 40:1) to afford **2** (415 mg, 88%) as a white solid.

LC-MS (ESI): m/z calcd for $C_{26}H_{31}N_8O_3Si^+$ $[M+H]^+$ 531.2, found 531.3.

^{1}H-NMR (500 MHz, $CDCl_3$): δ 8.31 (s, 1H), 8.04 (s, 1H), 7.58 (d, J = 7.3 Hz, 4H), 7.45 (dt, J = 7.6, 3.7 Hz, 2H), 7.38 (dt, J = 7.3, 3.7 Hz, 4H), 5.91 (d, J = 5.9 Hz, 1H), 5.68 (br, 2H), 4.95 (t, J = 5.8 Hz, 1H), 4.42 (dd, J = 5.7, 3.1 Hz, 1H), 4.26 (q, J = 3.3 Hz, 1H), 3.89 (dd, J = 11.7, 3.7 Hz, 1H), 3.80 (dd, J = 11.7, 3.1 Hz, 1H), 0.97 (s, 9H).

^{13}C-NMR (126 MHz, $CDCl_3$): *δ* 155.53, 152.56, 149.04, 138.93, 135.49, 135.48, 132.47, 132.36, 130.08, 130.03, 127.90, 127.87, 119.96, 90.26, 84.68, 77.28, 77.02, 76.77, 63.73, 62.65, 26.70, 19.11.

*9-[3-azido-3-deoxy-5-O-tert-butyldiphenylsily-2-O-(2,3,5-tri-O-acetyl-α-D-arabinofuranosyl)-β-D-ribofuranosyl]-adenine (**4**)*

Under a nitrogen atmosphere, **2** (493 mg, 1.55 mmol, 2.0 eq.) was dissolved in dichloroethane (DCE, 15 mL) and the resulting solution was cooled in an ice bath. $SnCl_4$ (1 M in DCM, 1.95 mL, 2.5 eq.) was added dropwise through a syringe. The reaction solution was stirred for 10 minutes before a solution of **3** (415 mg, 0.78 mmol, 1 eq.) in DCE (5 mL) was added, and the mixture was gradually warmed to room temperature. The reaction was complete after 24 hours. The resulting amber solution was treated with saturated $NaHCO_3$ (10 mL) and filtered through a pad of celite. The filtrate was partitioned between water (20 mL) and DCM (20 mL) and the organic phase was washed with brine, dried over anhydrous Na_2SO_4 and evaporated. The residue was purified by silica gel chromatography (DCM:MeOH 50:1) to afford **4** (500 mg, 81%) as a white solid.

LC-MS (ESI): m/z calcd for $C_{37}H_{45}N_8O_{10}Si^+$ $[M+H]^+$ 789.3, found 789.4.

^{1}H-NMR (500 MHz, $CDCl_3$): *δ* 8.25 (s, 1H), 8.07 (s, 1H), 7.66 (dd, *J* = 7.5, 4.3 Hz, 4H), 7.49 – 7.34 (m, 6H), 6.13 (d, *J* = 3.3 Hz, 1H), 5.76 (br, 2H), 5.31 (s, 1H), 5.19 (d, *J* = 1.9 Hz, 1H), 5.11 (dd, *J* = 5.6, 3.4 Hz, 1H), 5.03 (d, *J* = 4.8 Hz, 1H), 4.50 – 4.44 (m, 1H), 4.41 (dd, J = 11.9, 3.6 Hz, 1H), 4.36 (t, J = 6.2 Hz, 1H), 4.27 – 4.21 (m, 2H), 4.08 (dd, *J* = 12.0, 3.5 Hz, 1H), 3.82 (dd, *J* = 11.9, 3.2 Hz, 1H), 2.12 – 2.07 (m, 9H), 1.08 (s, 9H).

^{13}C-NMR (126 MHz, $CDCl_3$): 170.60, 170.29, 169.70, 155.36, 153.13, 149.40, 139.08, 135.63, 135.50, 132.56, 132.40, 130.09, 130.02, 127.93, 120.24, 105.74, 87.84, 82.08, 81.41, 81.34, 79.30, 77.29, 77.04, 76.79, 63.19, 62.65, 59.59, 26.89, 20.81, 20.73, 19.24.

*9-(3-azido-3-deoxy-5-O-tert-butyldiphenylsily-2-O-α-D-arabinofuranosyl-β-D-ribofuranosyl)-adenine (**5**)*

To a solution of **4** (500 mg, 0.63 mmol, 1 eq.) in MeOH (3 mL) was added K_2CO_3 (87 mg, 0.63

mmol, 1 eq.). The resulting suspension was stirred for 1 hour and directly evaporated to dryness and purified by column chromatography on silica gel (DCM:MeOH 40:1 to 20:1) to afford **5** (310 mg, 74%) as a white solid.

LC-MS (ESI): m/z calcd for $C_{31}H_{39}N_8O_7Si^+$ $[M+H]^+$ 663.3, found 663.3.

^{1}H-NMR (500 MHz, $CDCl_3$): δ 8.33 (s, 1H), 8.15 (s, 1H), 7.72 – 7.63 (m, 4H), 7.47 – 7.42 (m, 2H), 7.41 – 7.35 (m, 4H), 6.10 (d, J = 2.5 Hz, 1H), 5.78 (br, 2H), 5.41 (s, 1H), 5.01 (dd, J = 5.6, 2.6 Hz, 1H), 4.65 (br, overlapped, 1H), 4.62 (t, J = 6.5 Hz, 1H), 4.31 (s, 1H), 4.19 (dd, J = 6.6, 3.3 Hz, 1H), 4.17 – 4.06 (m, 3H), 3.91 (d, J = 12.5 Hz, 1H), 3.82 (dt, J = 11.9, 4.4 Hz, 2H), 3.60 – 3.38 (m, 1H), 1.09 (d, J = 2.1 Hz, 9H).

^{13}C-NMR (126 MHz, $CDCl_3$): δ 155.33, 153.19, 149.15, 138.80, 135.64, 135.44, 132.35, 132.17, 130.21, 130.09, 128.00, 120.15, 107.35, 88.21, 87.88, 82.60, 79.12, 78.10, 77.28, 77.03, 76.78, 62.12, 61.83, 59.88, 26.94, 19.23.

*9-(3-azido-3-deoxy-5-O-tert-butyldiphenylsily-2-O-[3,5-(1,1,3,3-tetraisopropyldisiloxane-1,3-diyl)-α-D-arabinofuranosyl]-β-D-ribofuranosy])-adenine (**6**)*

5 (310 mg, 0.47 mmol, 1 eq.) was dissolved in DMF (2 mL), followed by the addition of imidazole (143 mg, 2.10 mmol, 4.5 eq.) and $TIPDSCl_2$ (192 mg, 0.61 mmol, 1.3 eq.). The reaction mixture was allowed to stir overnight at room temperature before water (6 mL) was added. The mixture was extracted with ethyl acetate (5 mL * 3) and the combined organic phase was washed with brine, dried over Na_2SO_4 and evaporated to dryness. The residue was purified by column chromatography on silica gel (DCM:MeOH 40:1) to afford **6** as a white solid (272 mg, 64%).

LC-MS (ESI): m/z calcd for $C_{43}H_{65}N_8O_8Si_3^+$ $[M+H]^+$ 905.4, found 905.4.

^{1}H-NMR (500 MHz, $CDCl_3$): δ 8.17 (s, 1H), 7.98 (s, 1H), 7.67 (t, J = 7.3 Hz, 4H), 7.48 – 7.37 (m, 6H), 6.11 (d, J = 5.2 Hz, 1H), 5.72 (br, 2H), 5.11 (t, J = 5.4 Hz, 1H), 5.06 (d, J = 3.4 Hz, 1H), 4.38 (t, J = 5.1 Hz, 1H), 4.34 (dd, J = 7.4, 3.4 Hz, 1H), 4.26 – 4.16 (m, 3H), 4.08 – 4.01 (m, 2H), 4.08 – 3.98 (m, 2H) 3.81 (dd, J = 11.7, 3.4 Hz, 1H), 1.12 – 1.04 (m, 37H).

^{13}C-NMR (126 MHz, $CDCl_3$): δ 155.26, 153.05, 149.46, 147.88, 139.18, 135.61, 135.52, 132.65, 132.44, 130.07, 130.02, 127.94, 127.93, 119.94, 108.40, 86.94, 82.97, 81.41, 81.24,

80.13, 75.38, 63.28, 61.14, 60.89, 26.95, 19.26, 17.46, 17.33, 17.06, 17.03, 17.00, 13.50, 13.12, 12.82, 12.52.

9-(3-azido-3-deoxy-5-O-tert-butyldiphenylsily-2-O-[3,5-(1,1,3,3-tetraisopropyldisiloxane-1,3-diyl)-α-D-ribofuranosyl]-β-D-ribofuranosyl)-adenine (7)

6 (272 mg, 0.30 mmol, 1 eq.) in DMSO (2 mL) was treated with acetic anhydride (281 μL, 3.0 mmol, 10 eq.), and the reaction was stirred overnight at room temperature. Complete conversion of **6** was indicated by TLC. Ethanol (2 mL) was added to quench the reaction, followed by the addition of $NaBH_4$ (112 mg, 3.0 mmol, 10 eq.). The resulting mixture was stirred for 1 hour before being quenched with acetone (2 mL). The reaction mixture was directly evaporated to dryness and the residue was purified on silica gel column (DCM:MeOH 40:1) to afford **7** as a colorless syrup (168 mg, 62%, two steps).

LC-MS (ESI): m/z calcd for $C_{43}H_{65}N_8O_8Si_3^+$ $[M+H]^+$ 905.4, found 905.4.

^{1}H-NMR (500 MHz, $CDCl_3$): *δ* 8.29 (s, 1H), 8.09 (s, 1H), 7.68 (t, J = 5.8 Hz, 4H), 7.49 – 7.44 (m, 2H), 7.49 – 7.37 (m, 4H), 6.19 (d, J = 4.1 Hz, 1H), 5.58 (br, 2H), 5.19 (d, J = 4.2 Hz, 1H), 5.14 (t, J = 4.9 Hz, 1H), 4.45 (t, J = 5.6 Hz, 1H), 4.29 – 4.20 (m, 3H), 4.12 – 4.01 (m, 3H), 3.83 (dd, J = 11.8, 3.3 Hz, 1H), 3.72 (dd, J = 11.9, 8.2 Hz, 1H), 3.17 (d, J = 9.1 Hz, 1H), 1.12 – 1.06 (m, 37H).

^{13}C-NMR (126 MHz, $CDCl_3$): δ 155.32, 153.19, 139.08, 135.63, 135.49, 132.54, 132.42, 130.08, 130.01, 127.93, 101.70, 87.46, 84.34, 82.45, 79.22, 71.14, 70.84, 63.88, 62.91, 60.45, 53.43, 26.92, 19.23, 17.51, 17.41, 17.38, 17.35, 17.10, 16.96, 16.88, 16.79, 13.48, 13.37, 13.07, 12.49.

*9-(3-azido-3-deoxy-2-O-α-D-ribofuranosyl-β-D-ribofuranosyl)-adenine (**8**)*

To a stirring solution of **7** (30 mg, 0.033 mmol, 1 eq.) in THF (300 μL) was added TBAF (1 M in THF, 100 μL, 3 eq.) and the mixture was stirred for 1 hour at room temperature. Dowex-50WX8 cation exchange resin (100 mg, NH_4^+ form) was added and the resulting suspension was stirred for 30 minutes. The resin was filtered off and the filtrate was concentrated and purified by column chromatography on silica gel (DCM:MeOH 10:1) to afford **8** as a white

solid (12 mg, 86%).

LC-MS (ESI): m/z calcd for $C_{15}H_{21}N_8O_7Si^+$ $[M+H]^+$ 425.2, found 425.2.

^{1}H NMR (500 MHz, D_2O) δ 8.32 (s, 1H), 8.23 (s, 1H), 6.16 (d, *J* = 6.1 Hz, 1H), 5.11 (d, *J* = 4.2 Hz, 1H), 5.04 (t, *J* = 6.0 Hz, 1H), 4.58 (dd, *J* = 5.7, 3.7 Hz, 1H), 4.37 (q, *J* = 3.2 Hz, 1H), 4.24 (dt, *J* = 4.6, 3.2 Hz, 1H), 4.03 (qd, *J* = 6.3, 3.7 Hz, 2H), 3.92 (dd, *J* = 13.0, 2.7 Hz, 1H), 3.84 (dd, *J* = 13.0, 3.4 Hz, 1H), 3.69 (dd, *J* = 12.5, 3.3 Hz, 1H), 3.62 (dd, *J* = 12.5, 4.6 Hz, 1H).

^{13}C-NMR (126 MHz, D_2O): δ 155.85, 152.77, 148.54, 140.84, 119.31, 103.26, 87.14, 85.23, 84.20, 79.90, 71.22, 69.51, 61.96, 61.59, 61.39.

*3'-azido-3'-deoxy-iso-ADP-ribose (**9**)*

A solution of **8** (12 mg, 0.028 mmol, 1 eq.) in trimethyl phosphate (280 µL) was cooled to 0 ºC in an ice bath. $POCl_3$ (25.6 µL, 0.28 mmol, 10 eq.) was added slowly into the reaction mixture and the reaction was allowed to proceed for 6 hours at 0 ºC. TLC and LC-MS analysis indicated the starting material was mainly converted into the desired diphosphate with two monophosphates as minor side products and traces of triphosphates were also formed. The reaction was quenched with ice-cold water, washed with DCM to remove trimethyl phosphate, and neutralized with ammonium hydroxide to pH 10. The resulting solution was evaporated to dryness and the residue was purified on silica gel column (iPrOH:H_2O:NH_4OH 6:1:1 to 3:1:1) followed by size-exclusion chromatography purification on HW-40 resin (100% H_2O) to afford **9** as a white solid (5 mg, 31%).

HRMS (ESI): m/z calcd for $C_{15}H_{23}N_8O_{13}P_2^+$ $[M+H]^+$ 585.0854, found 585.0846.

^{1}H-NMR: (500 MHz, D_2O) δ 8.57 (s, 1H), 8.22 (s, 1H), 6.21 (d, J = 5.6 Hz, 1H), 5.24 (d, J = 3.3 Hz, 1H), 5.00 (t, J = 5.7 Hz, 1H), 4.63 (t, J = 4.8 Hz, 1H), 4.44 (s, 1H), 4.36 (s, 1H), 4.17 – 4.12 (d, J = 2.7 Hz, 2H), 4.07 – 3.97 (m, 2H), 3.88 – 3.76 (m, 2H).

^{13}C-NMR: (126 MHz, D_2O) δ 155.52, 152.77, 149.00, 139.92, 118.71, 103.27, 85.95, 84.39, 84.33, 82.79, 82.72, 80.89, 71.17, 69.72, 64.48, 64.30, 61.81.

^{31}P-NMR: (202 MHz, D_2O) δ 2.39, 1.22.

3'-deoxy-3'-(4-(3-((6-(2',7'-bis(dimethylamino)-3-oxo-3H-spiro[isobenzofuran-1,9'-

*xanthene]-5-carboxamido)hexyl)amino)-3-oxopropyl)-1H-1,2,3-triazol-1-yl)-iso-ADP-ribose **(10)***

To a stirring solution of azide **9** (3.3 mg, 5.6 μmol, 1 eq.) in water (100 μL) was added $CuSO_4$ (0.72 mg, 4.5 μmol, 0.8 eq.), sodium ascorbate (1.8 mg, 9.0 μmol, 1.6 eq.), $NaHCO_3$ (0.94 mg, 11.2 μmol, 2 eq.) and **TAMRA-alkyne** (3.4 mg, 5.6 μmol, 1 eq.) in *t*BuOH (100 μL). The reaction was allowed to proceed at room temperature for 2 hours before complete conversion of the azide was observed by LC-MS. The reaction mixture was directly purified on a size-exclusion chromatography column (HW-40 resin, 0.15 M NH_4OAc). Fractions containing the desired product were pooled and lyophilized repeatedly. The crude sample was then subjected to preparative HPLC purification, yielding **TAMRA-isoADPr** (1.5 mg, 22%) as a dark purple solid. It should be noted that the purified sample showed extensive peak broadening in NMR, possibly due to ring-opening isomerization of the TAMRA structure catalyzed by the acidic phosphate groups. Therefore, the sample was passed through a short column of Dowex 50WX8 ion-exchange resin (NH_4^+ form) to give the ammonium salt of **TAMRA-isoADPr** with improved NMR peak shapes.

HRMS (ESI): m/z calcd for $C_{51}H_{63}N_{12}O_{18}P_2^+$ $[M+H]^+$ 1193.3853, found 1193.3866.

^{1}H NMR (500 MHz, D_2O) δ 8.44 – 8.31 (m, 1H), 8.23 (s, 1H), 8.05 (d, J = 7.9 Hz, 1H), 7.97 (s, 1H), 7.83 (s, 1H), 7.46 (d, J = 9.2 Hz, 1H), 7.09 (d, J = 10.2 Hz, 1H), 6.96 (d, J = 9.2 Hz, 1H), 6.71 (d, J = 9.4 Hz, 1H), 6.64 (d, J = 9.3 Hz, 1H), 6.29 – 6.13 (m, 3H), 5.59 (d, J = 6.3 Hz, 1H), 4.99 (d, J = 5.4 Hz, 1H), 4.90 (d, J = 6.5 Hz, 1H), 4.66 (s, 1H), 4.00 (s, 2H), 3.92 (t, J = 5.6 Hz, 1H), 3.87 (dd, J = 6.5, 2.8 Hz, 1H), 3.79 – 3.71 (m, 2H), 3.48 – 3.36 (m, 3H), 3.21 – 3.11 (m, 2H), 3.11 – 3.06 (m, 2H), 3.05 – 2.99 (m, 12H), 2.70 – 2.50 (m, 2H), 1.68 – 1.54 (m, 2H), 1.42 – 1.29 (m, 4H), 1.26 – 1.12 (m, 2H).

^{13}C NMR (126 MHz, D_2O) δ 174.7, 157.5, 156.7, 156.6, 156.6, 148.2, 146.1, 137.0, 135.4, 134.2, 130.8, 125.2, 120.6, 118.9, 113.7, 112.6, 103.4, 95.9, 86.9, 84.3, 81.5, 72.4, 70.8, 69.0, 64.7, 64.4, 61.1, 40.0, 39.1, 35.3, 28.2, 28.1, 25.5, 21.3.

^{31}P-NMR: (202 MHz, D_2O) δ 0.14, -0.04.

*3'-deoxy-3'-(4-(biotin-PEG4)-1H-1,2,3-triazol-1-yl)-iso-ADP-ribose **(11)***

Azide **9** (30 µL, 30 mg/mL, 1 eq.) in water was mixed with commercially available Biotin-PEG4-alkyne (70 µL, 10 mg/mL, 1 eq.) in DMF, followed by the addition of $CuSO_4$ (20 µL, 10 mg/mL), sodium ascorbate (24 µL, 20 mg/mL) and 4 µL saturated $NaHCO_4$. After completion, the reaction mixture was directly purified by preparative HPLC. The desired fraction was lyophilized and redissolved in 50 µL water. The molar concentration of the obtained **biotin-isoADPr** stock solution was estimated by LC-MS using 3'-azido-isoADPr as an external standard and was directly used in biolayer interferometry experiments.

LC-MS (ESI): m/z calcd for $C_{36}H_{58}N_{11}O_{19}P_2S^+$ $[M+H]^+$ 1042.3, found 1042.4.

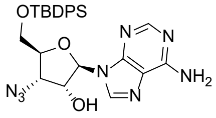

2

^{1}H-NMR

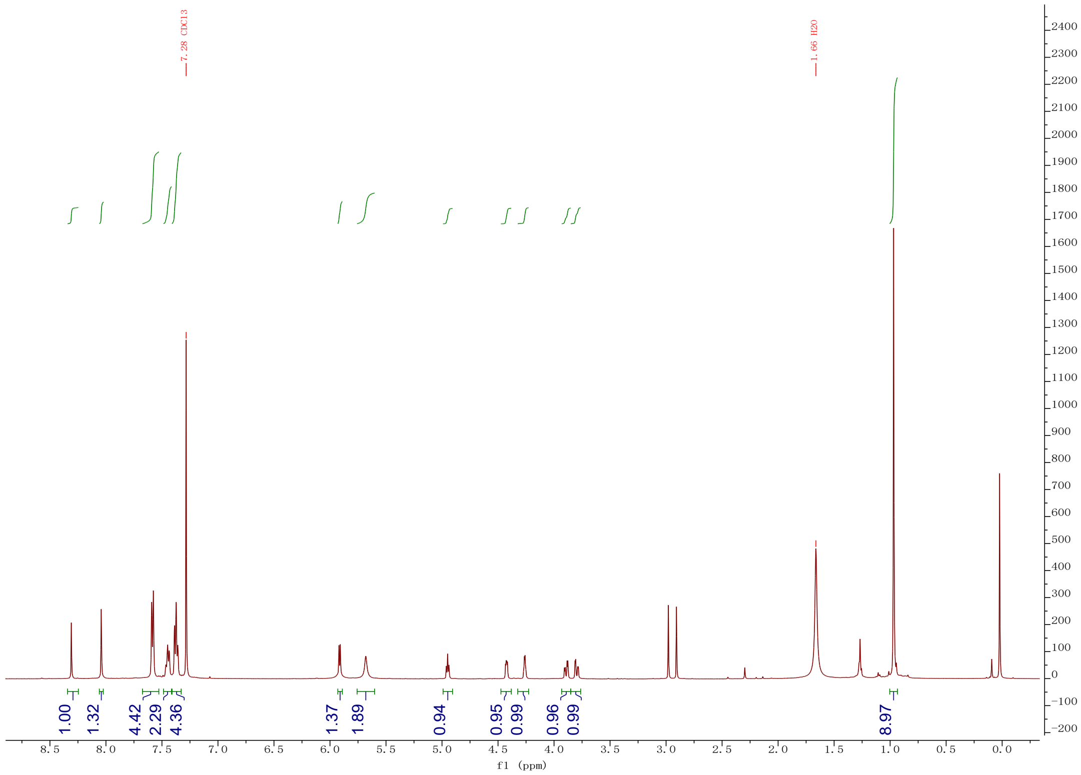

^{13}C-NMR

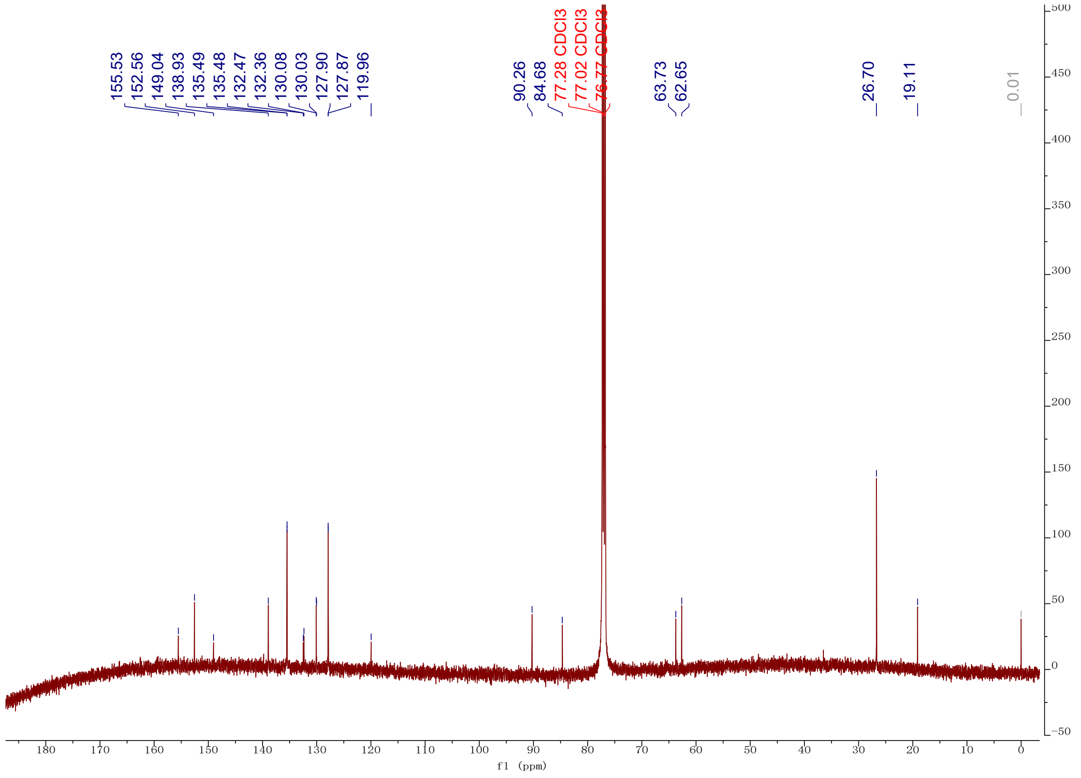

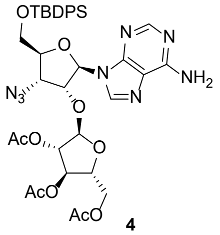

^{1}H-NMR

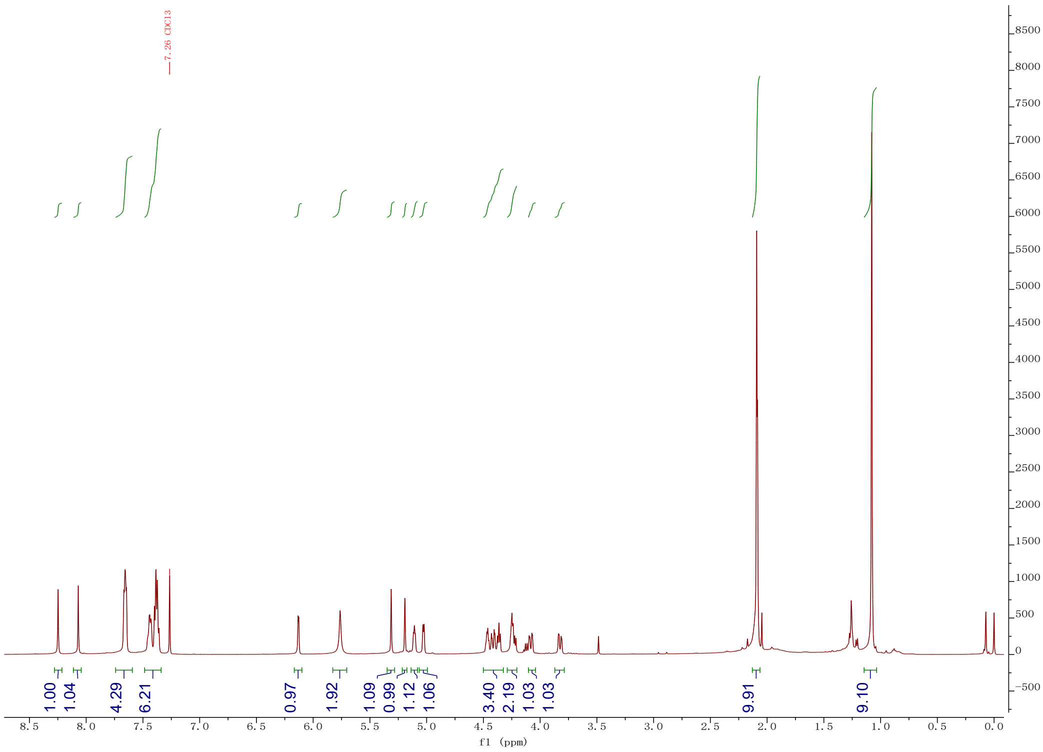

^{13}C-NMR

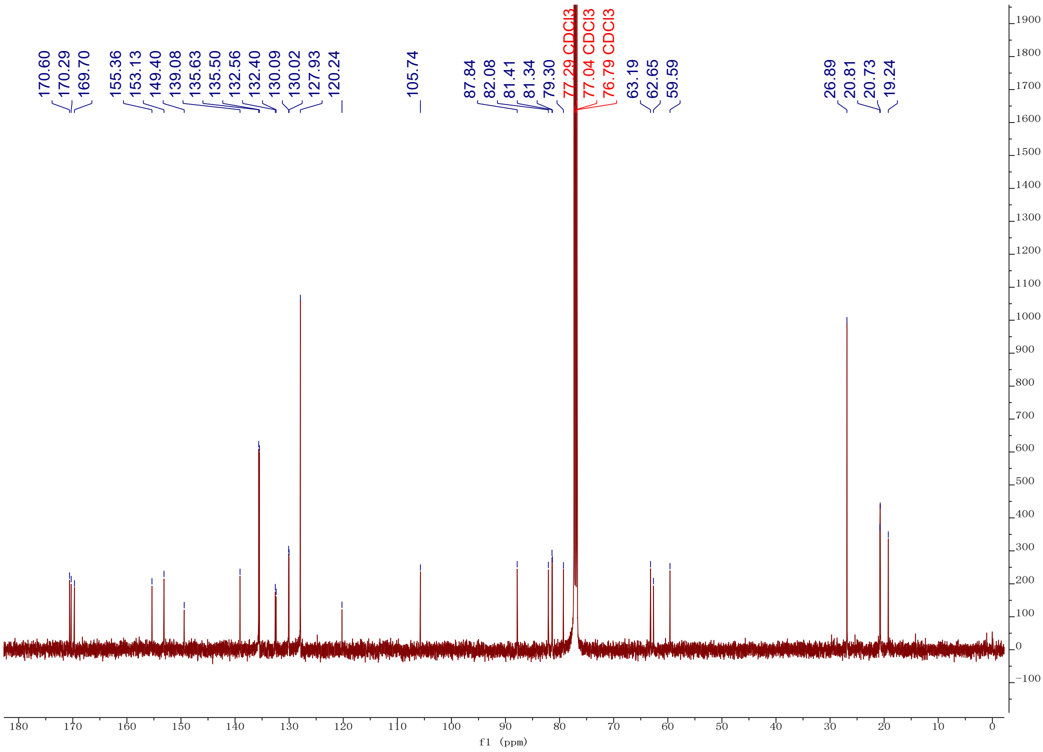

COSY

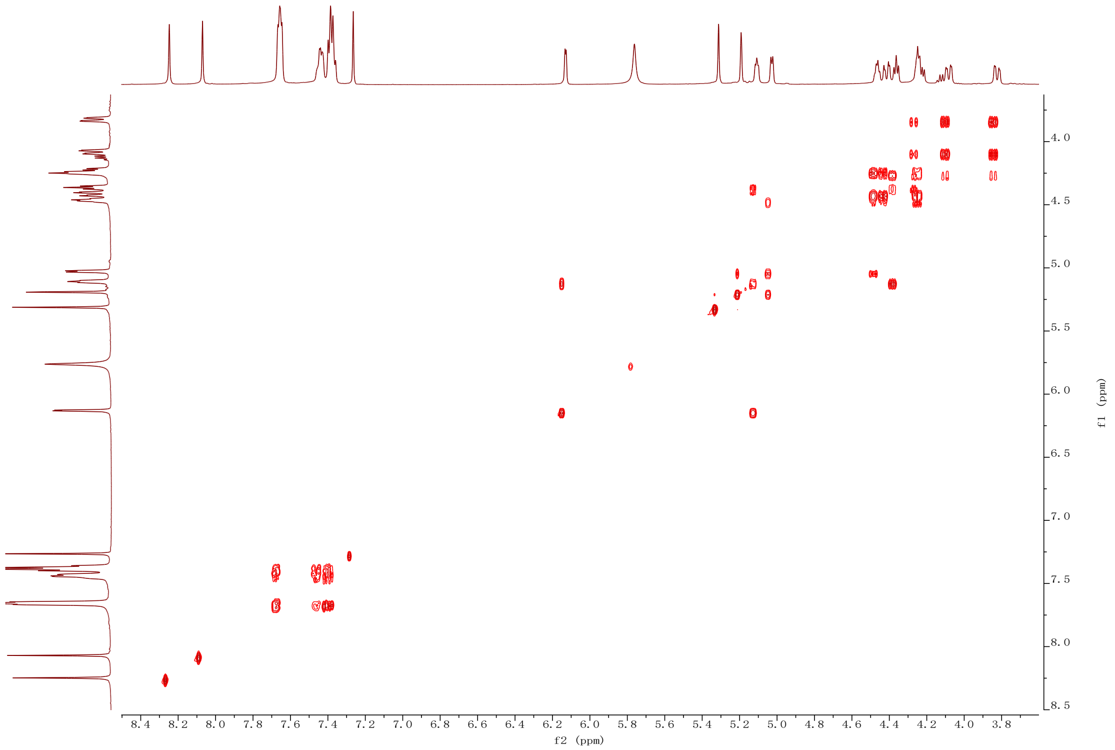

HSQC

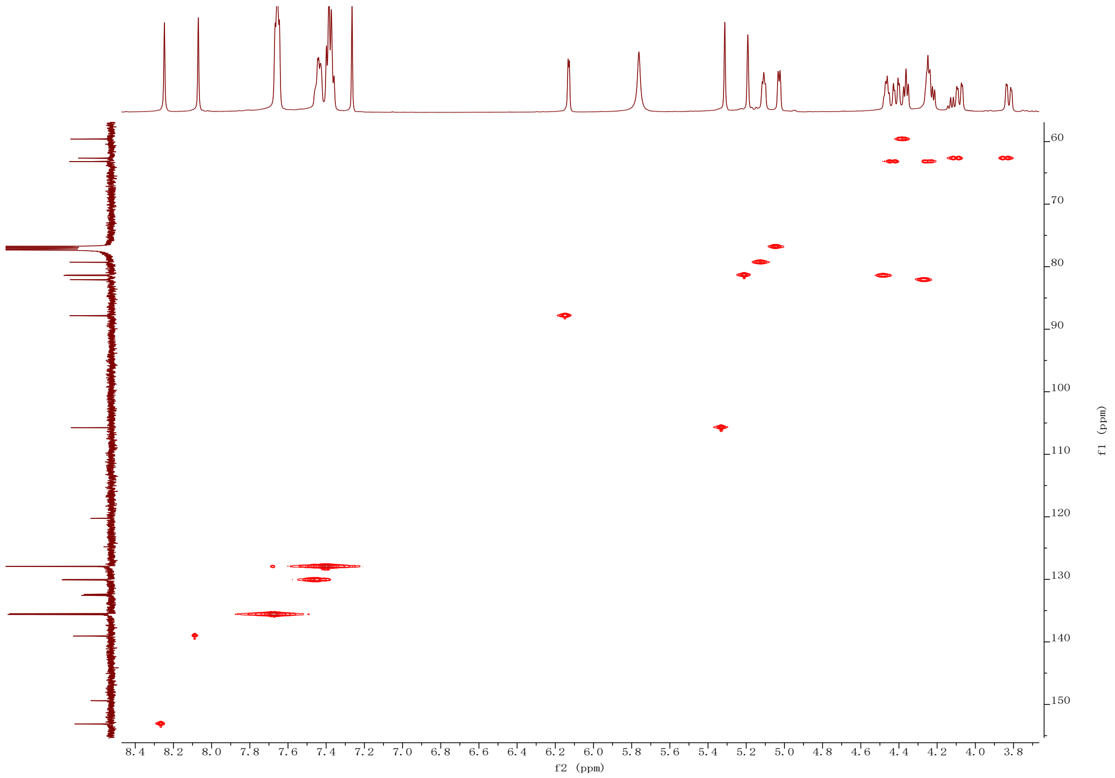

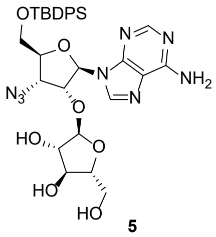

5

^{1}H-NMR

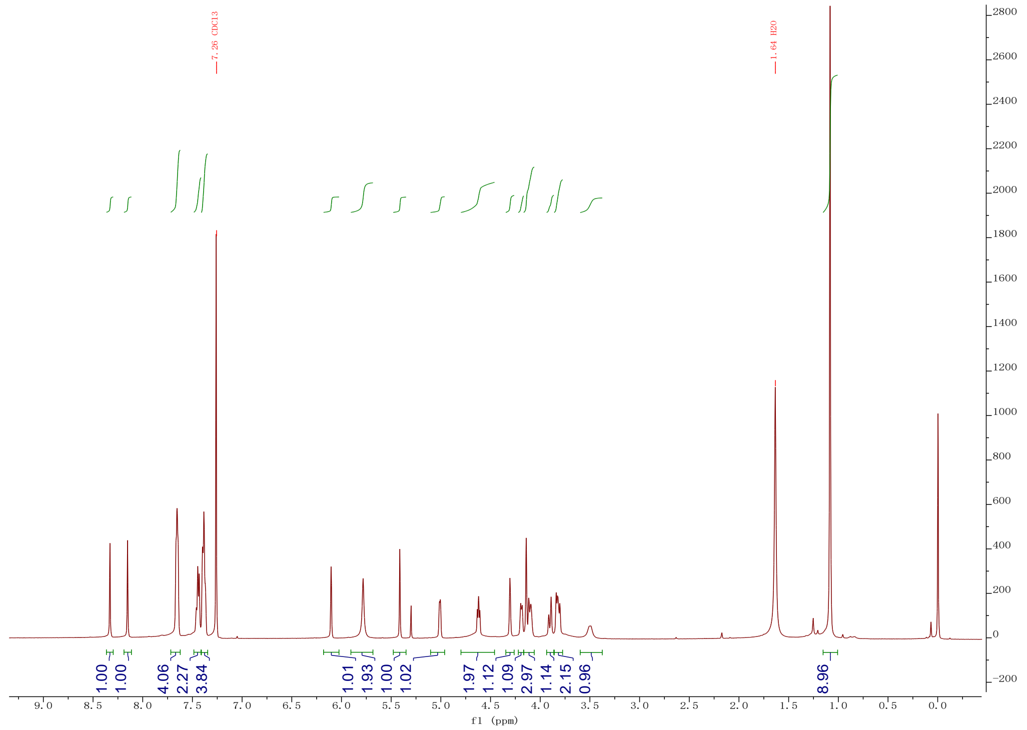

^{13}C-NMR

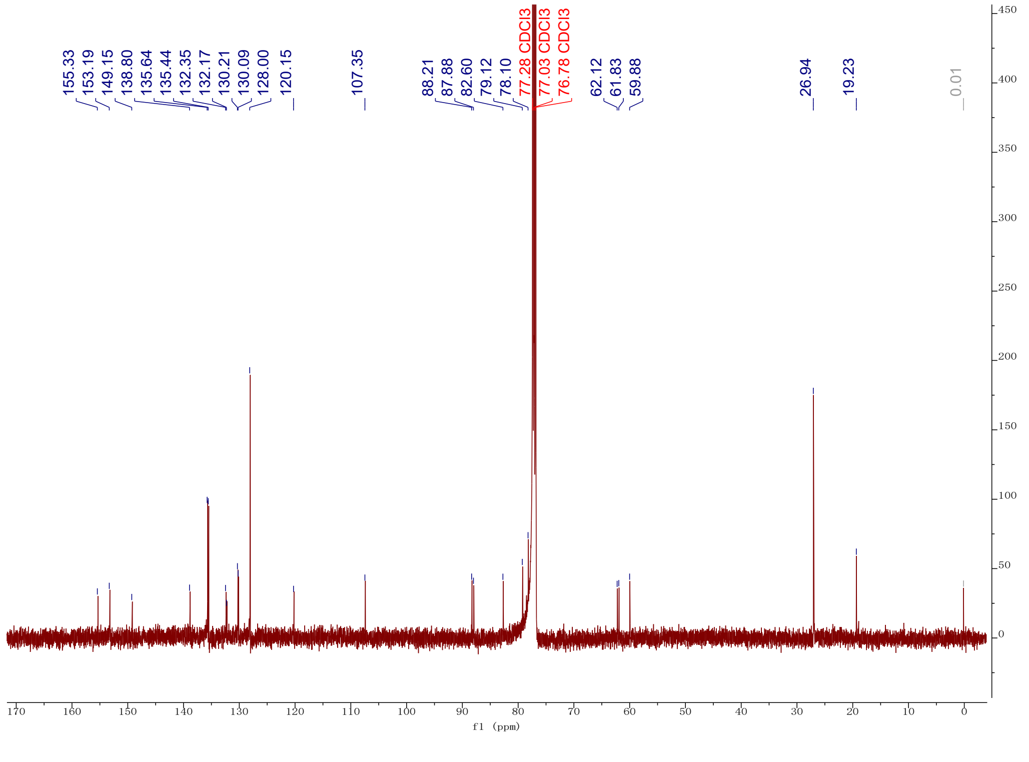

COSY

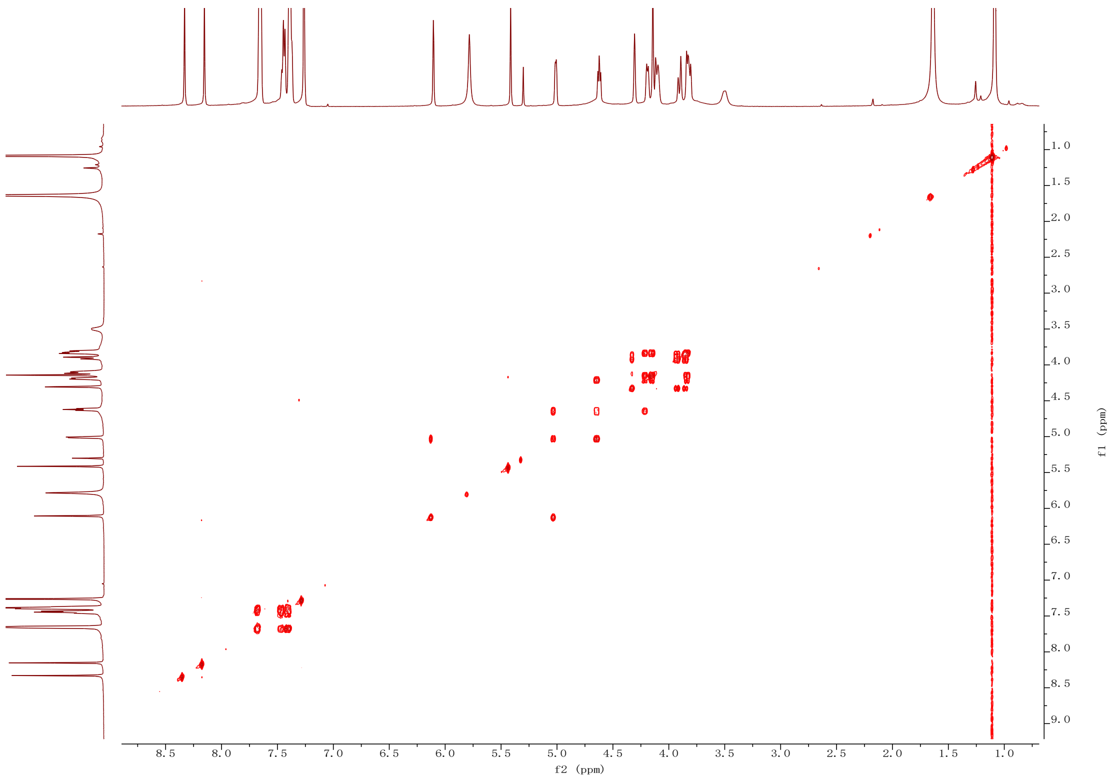

HSQC

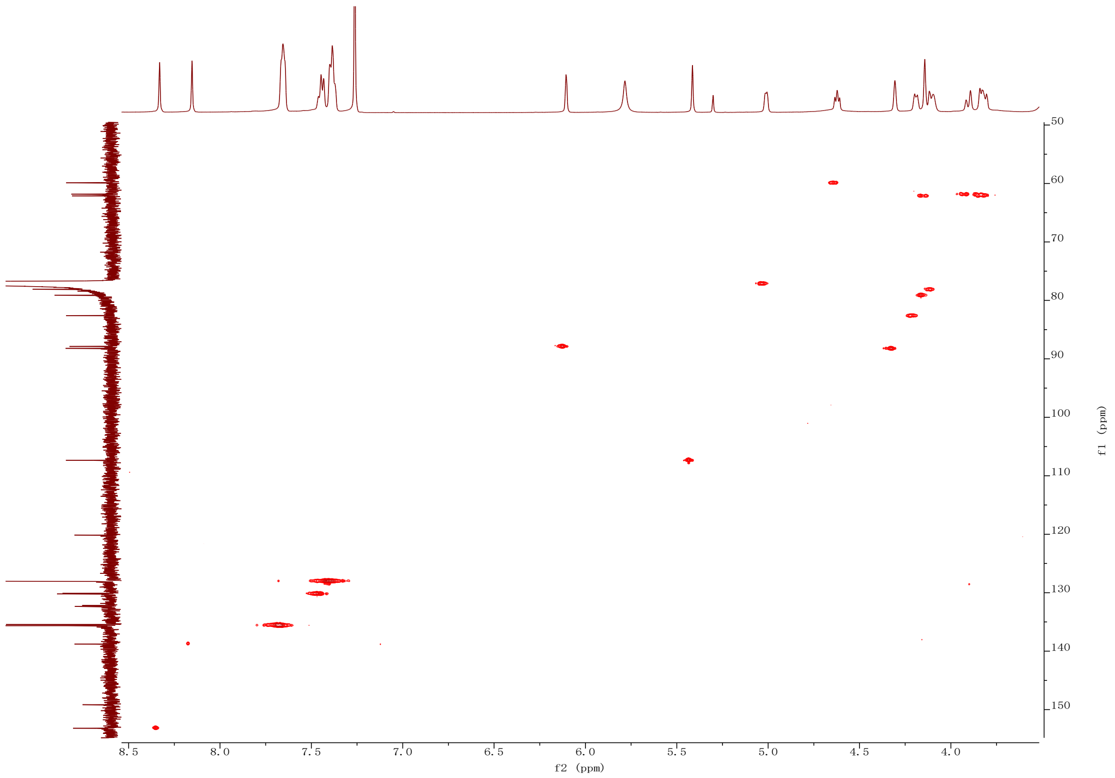

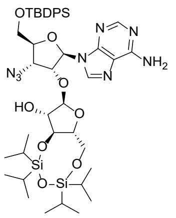

6

^{1}H-NMR

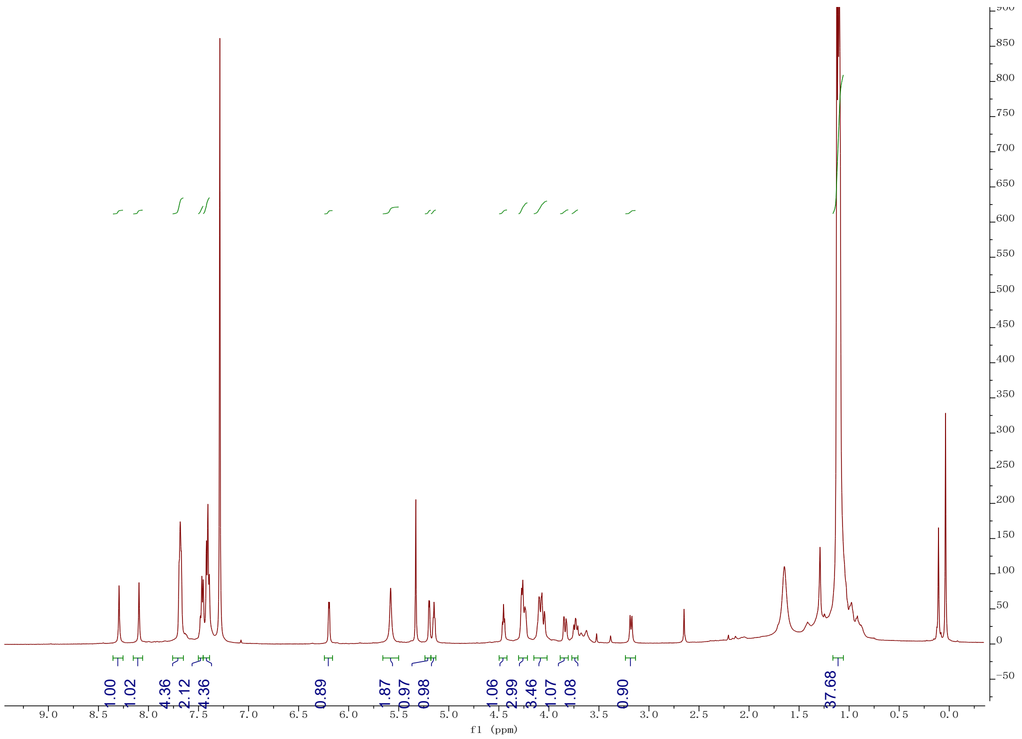

^{13}C-NMR

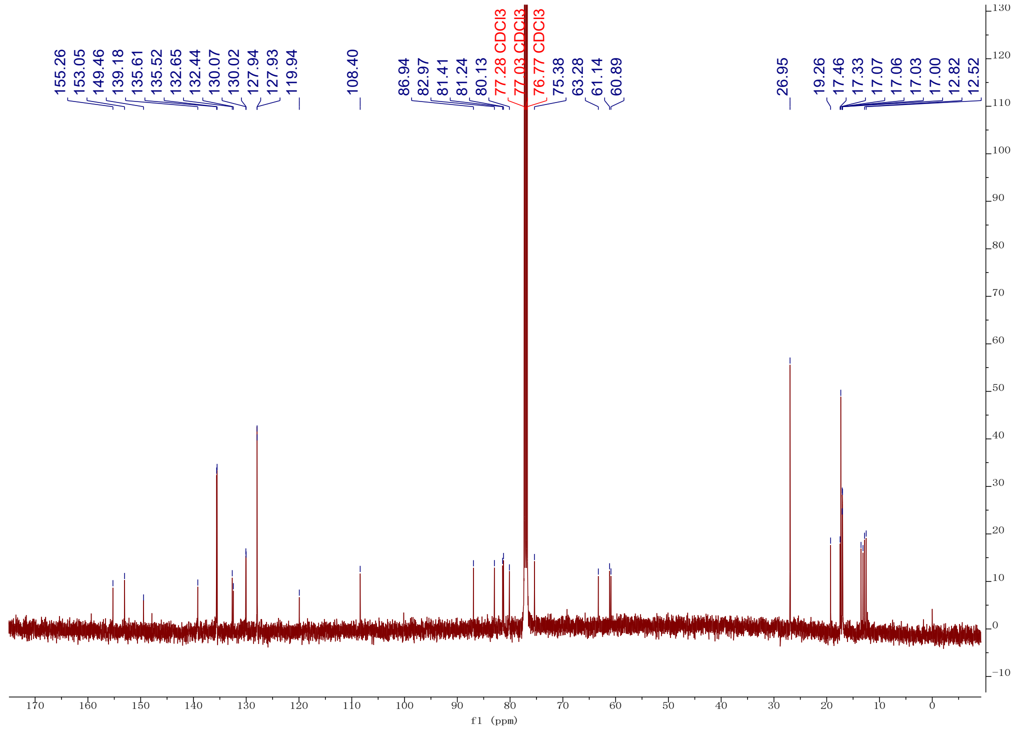

COSY

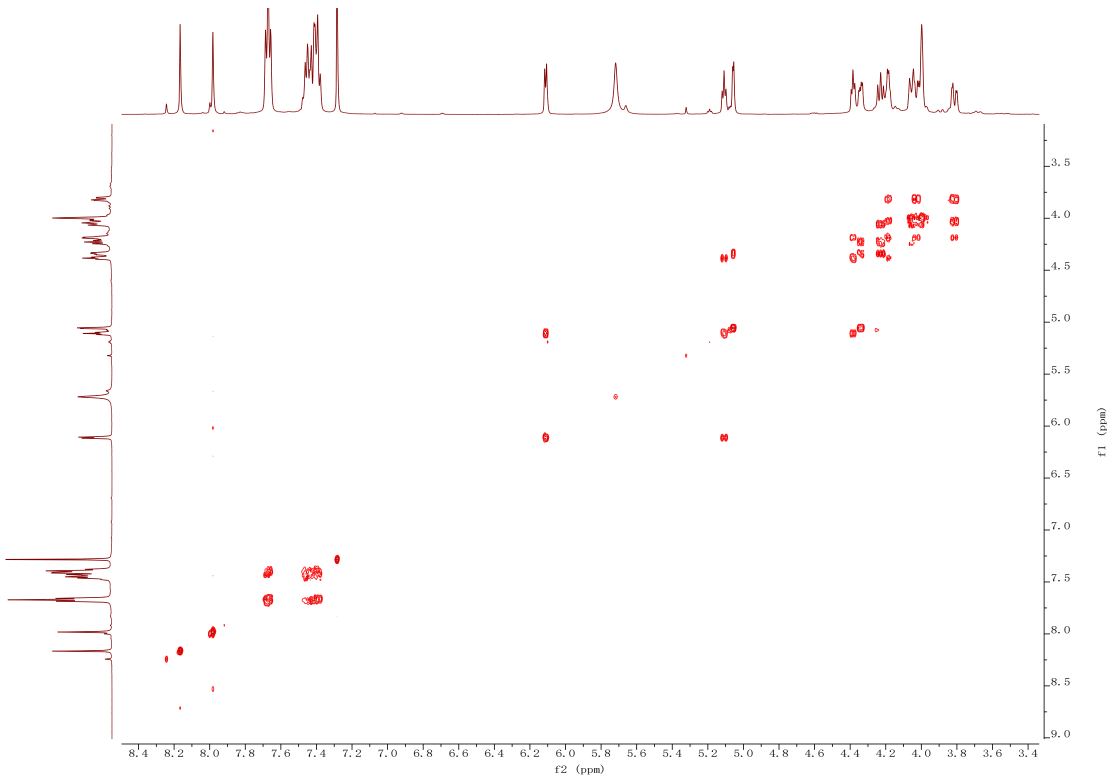
8.4 8.2 8.0 7.8 7.6 7.4 7.2 7.0 6.8 6.6 6.4 6.2 6.0 5.8 5.6 5.4 5.2 5.0 4.8 4.6 4.4 4.2 4.0 3.8 3.6 3.4
f2 (ppm)
3.5 4.0 4.5 5.0 5.5 6.0 6.5 7.0 7.5 8.0 8.5 9.0
f1 (ppm)

HSQC

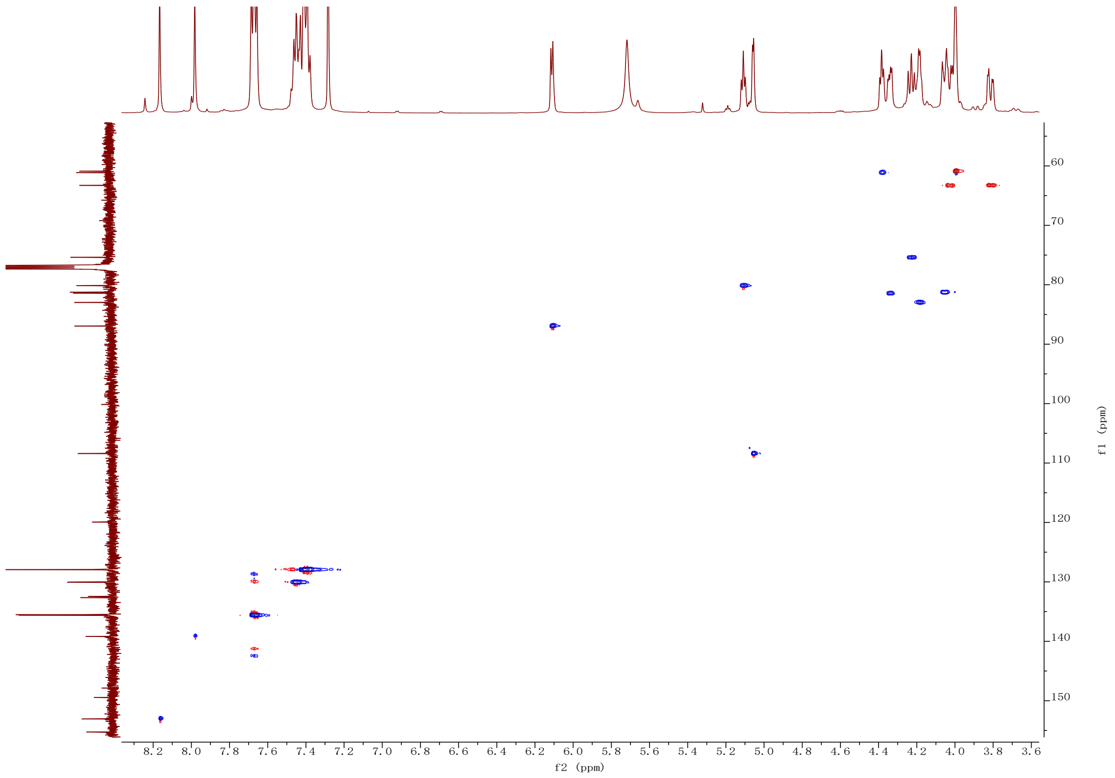
8.2 8.0 7.8 7.6 7.4 7.2 7.0 6.8 6.6 6.4 6.2 6.0 5.8 5.6 5.4 5.2 5.0 4.8 4.6 4.4 4.2 4.0 3.8 3.6
f2 (ppm)
60 70 80 90 100 110 120 130 140 150
f1 (ppm)

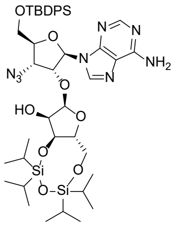

7

^{1}H-NMR

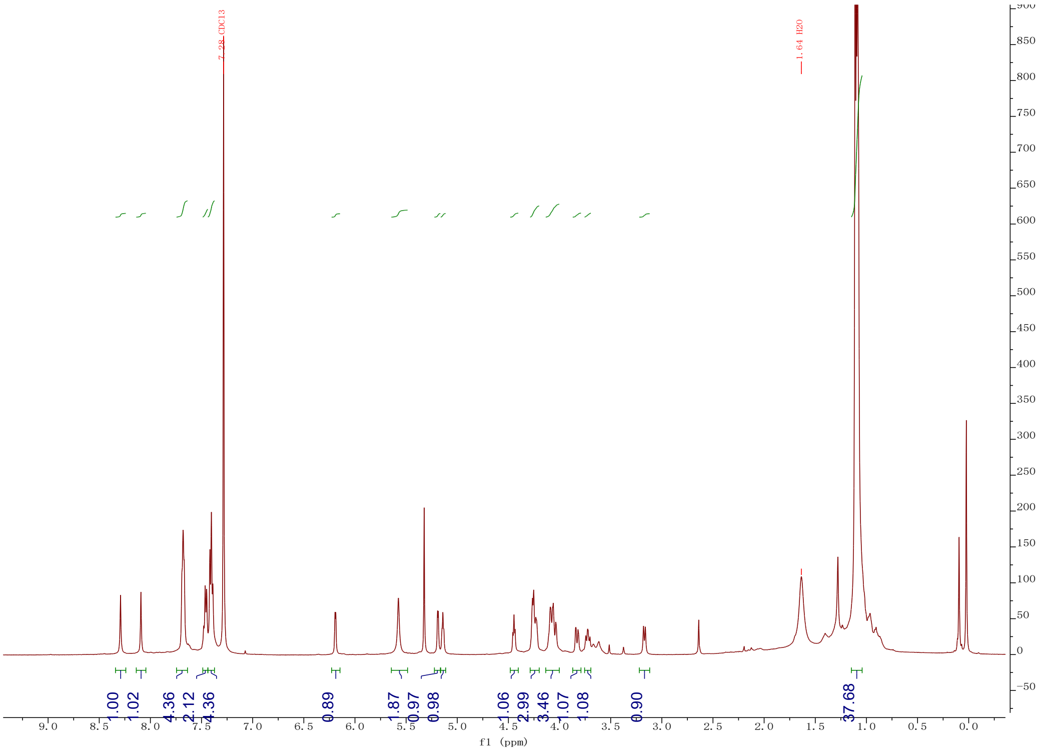

^{13}C-NMR

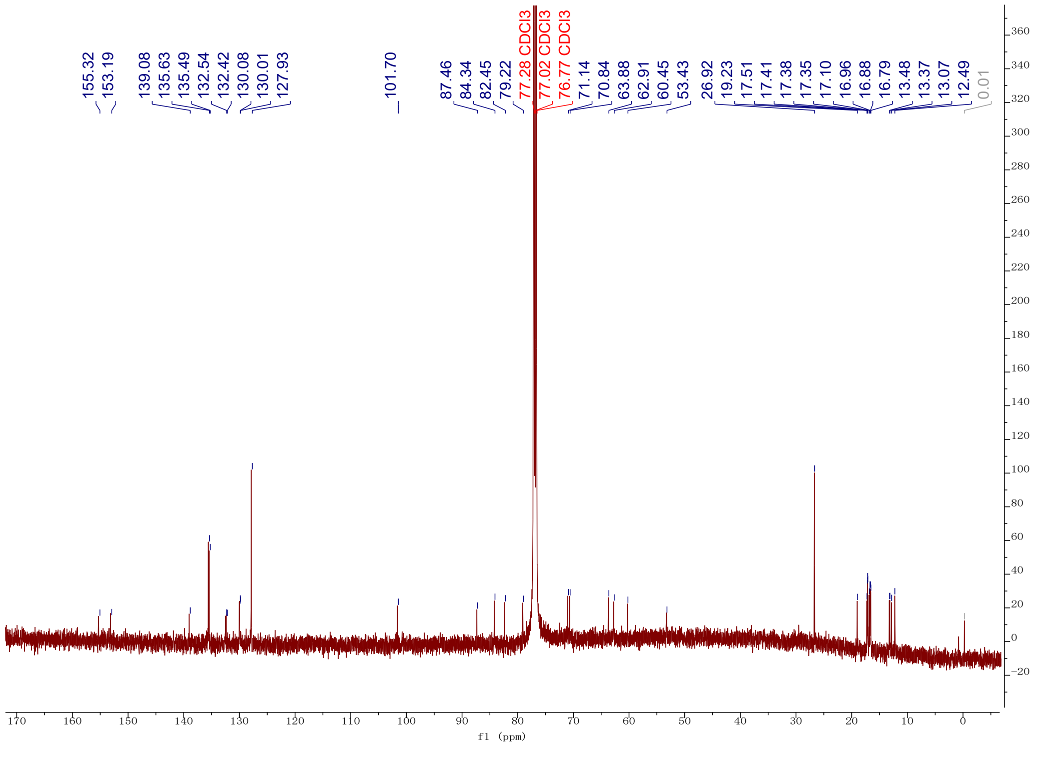

COSY

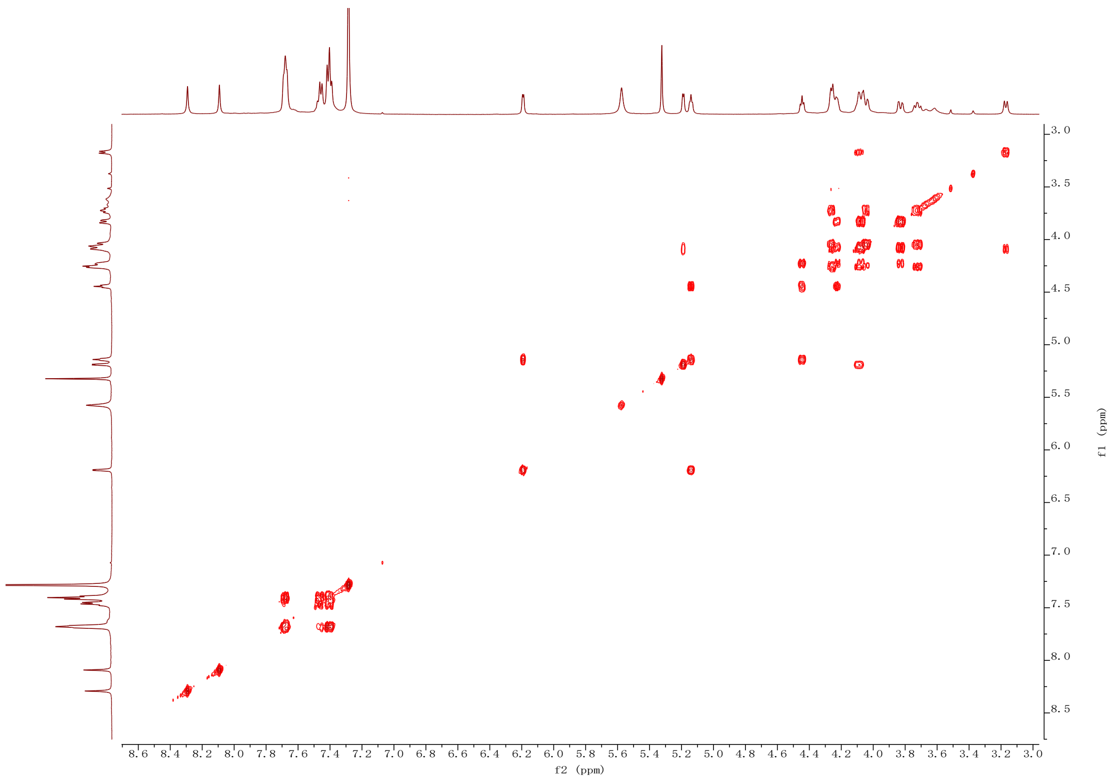

HSQC

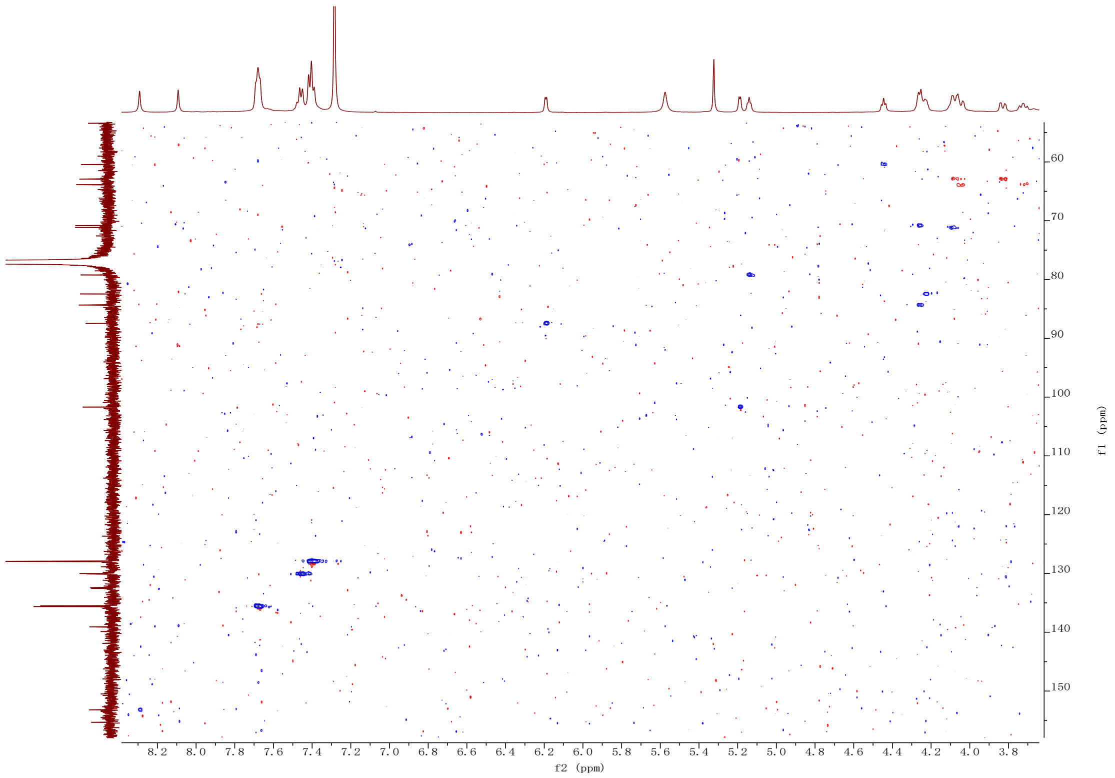

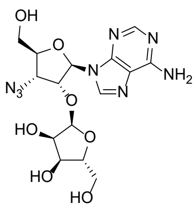

8

^{1}H-NMR

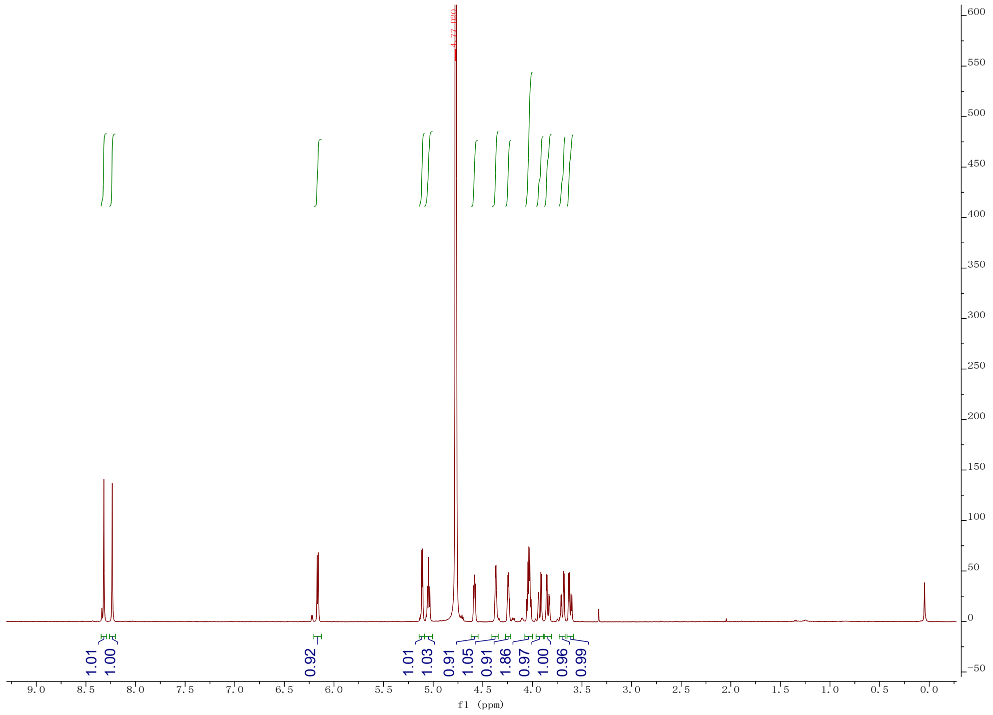

^{13}C-NMR

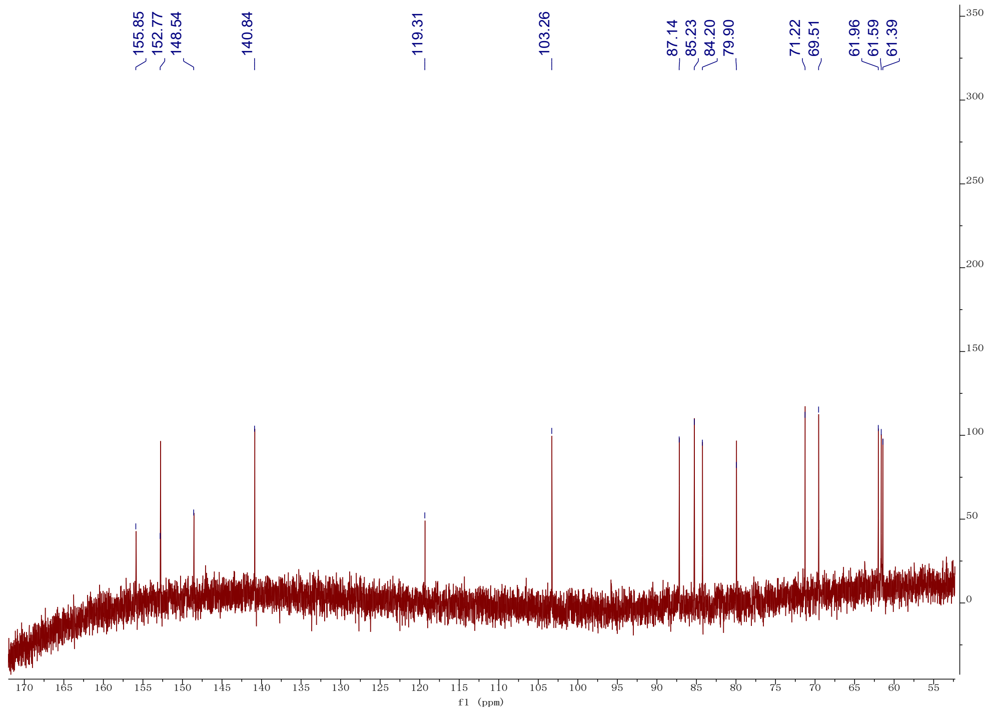

COSY

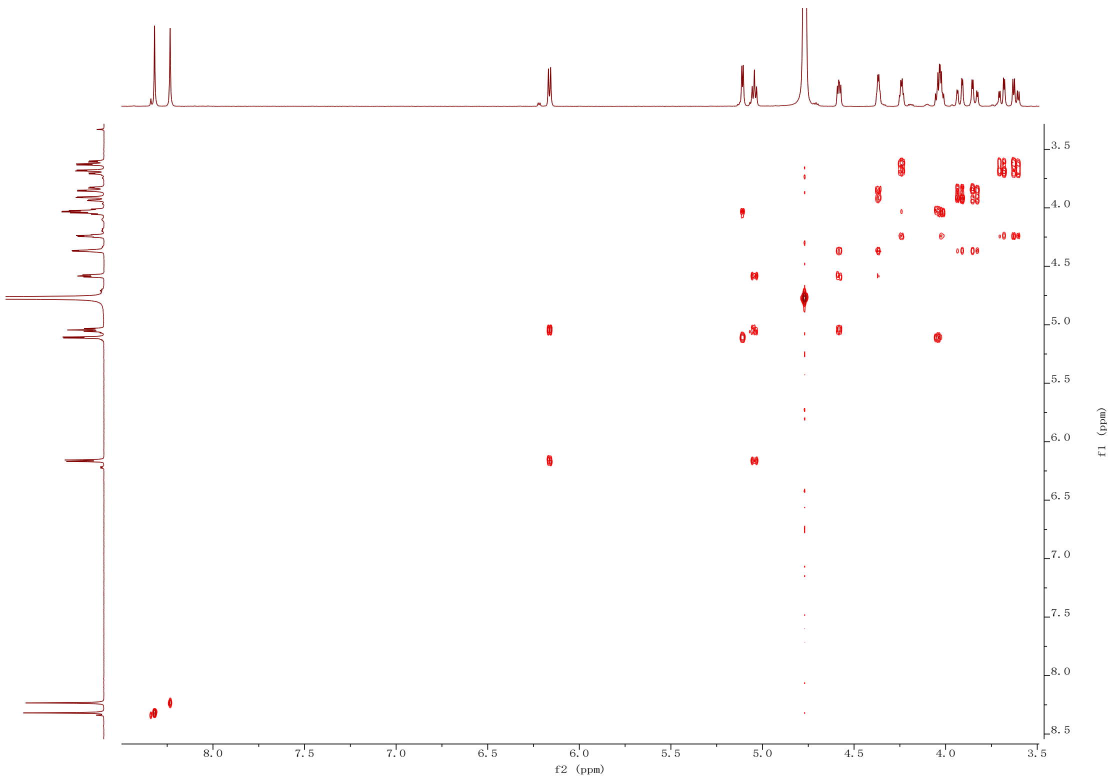

HSQC

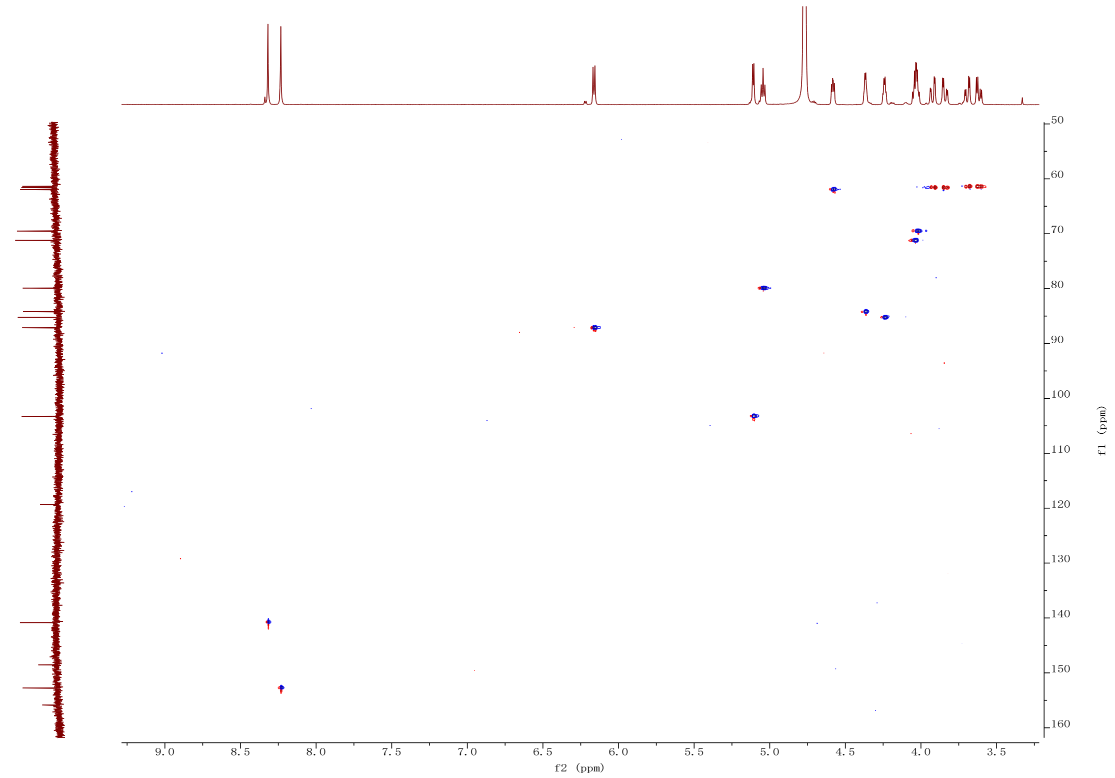

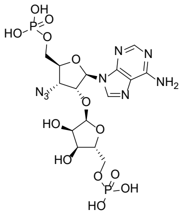

9

^{1}H-NMR

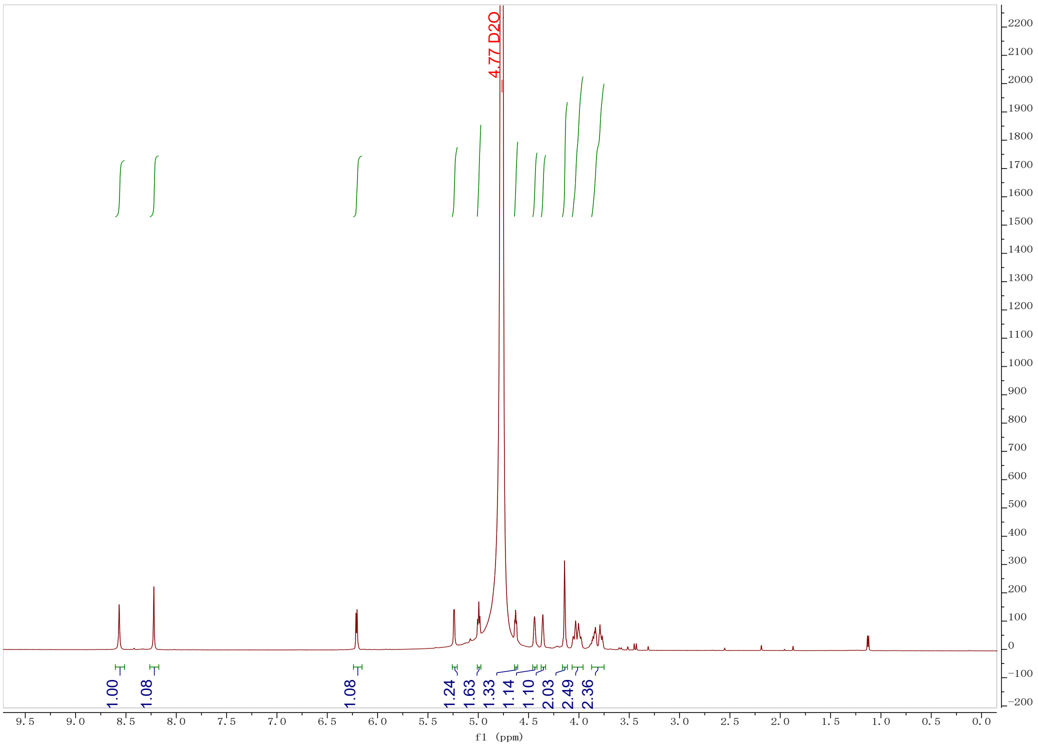

^{13}C-NMR

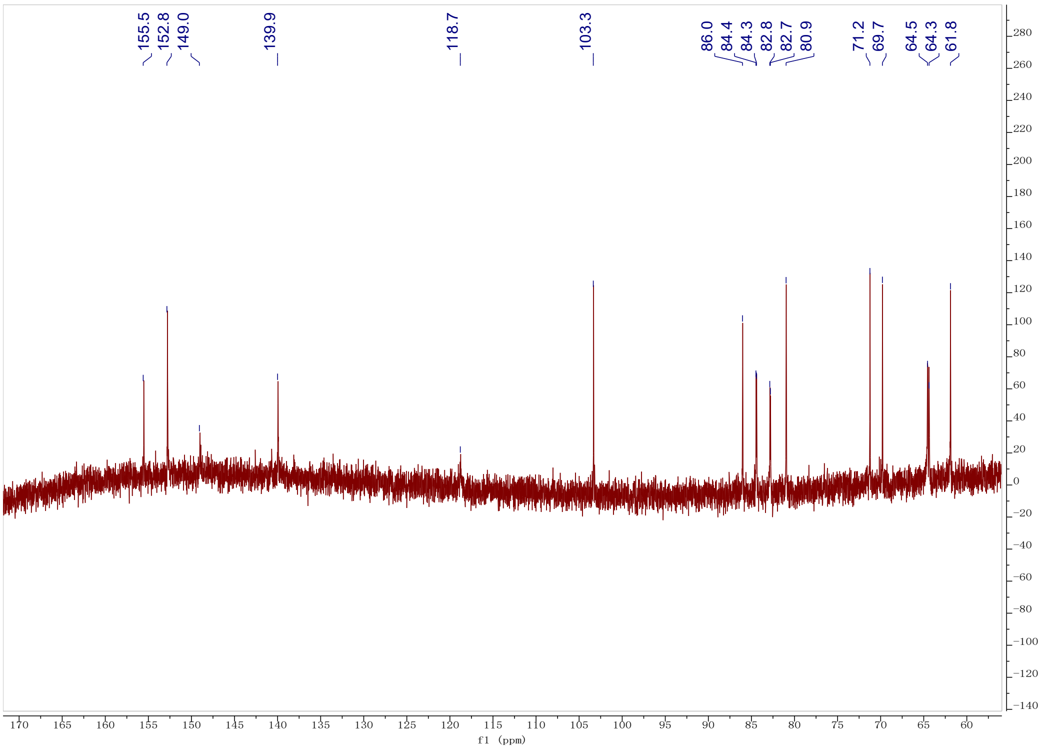

^{31}P-NMR

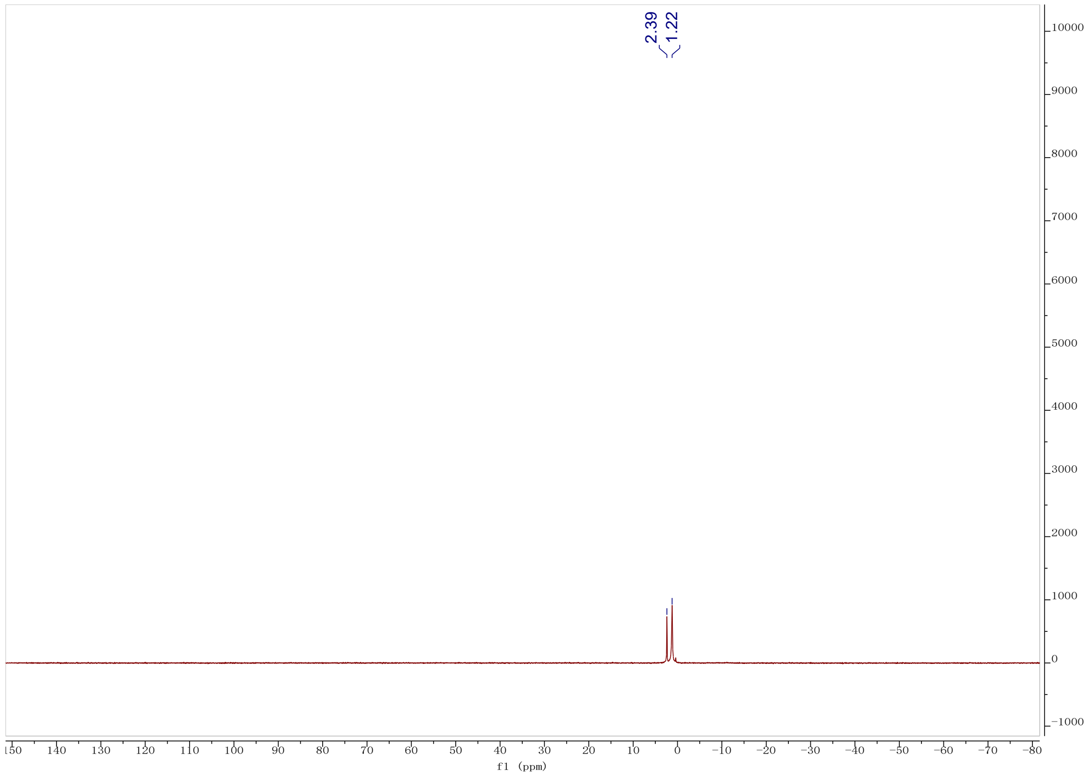

COSY

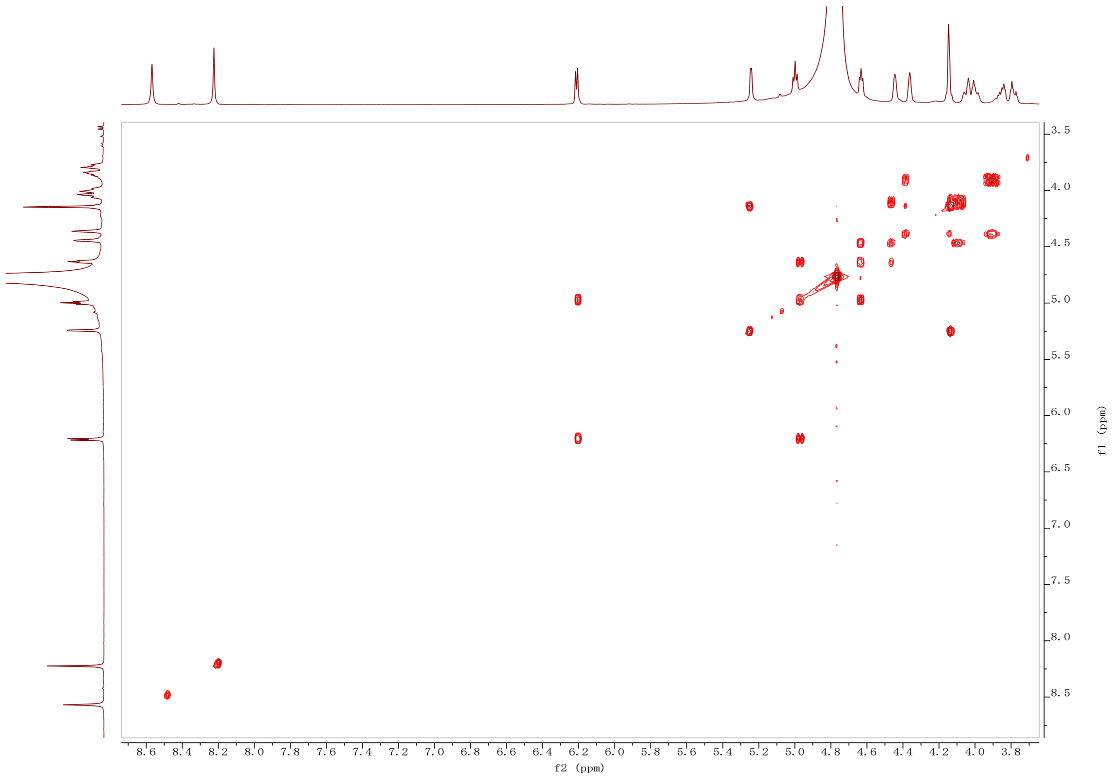

HSQC

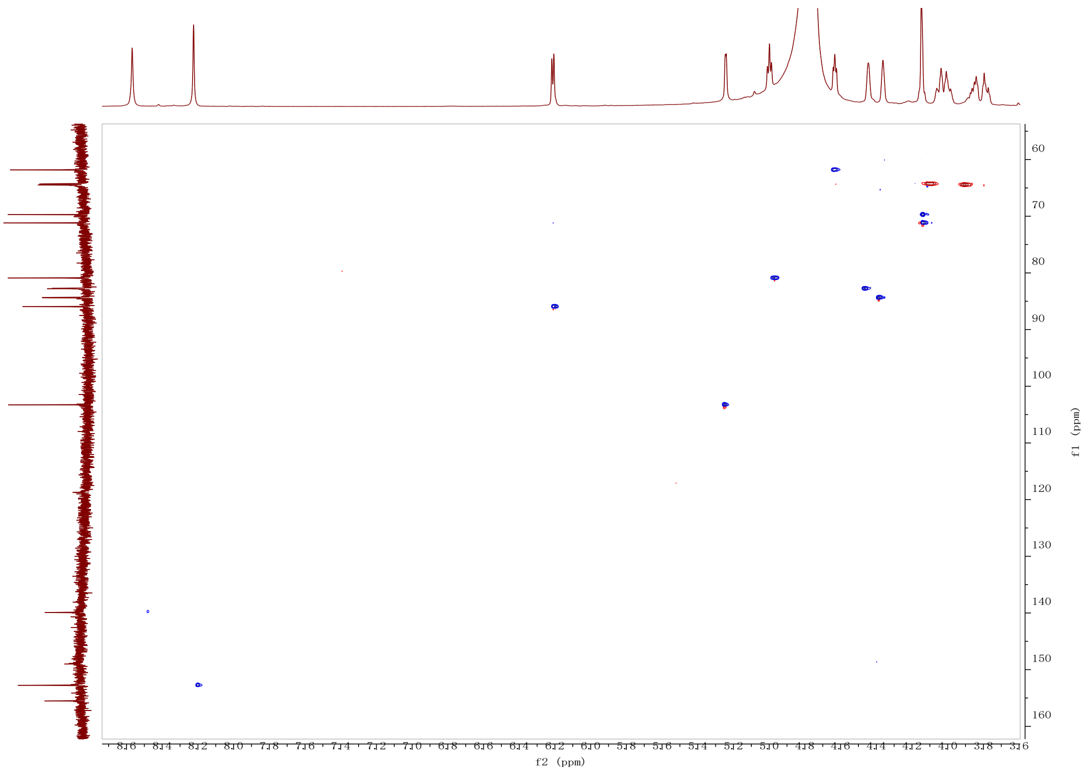

HRMS

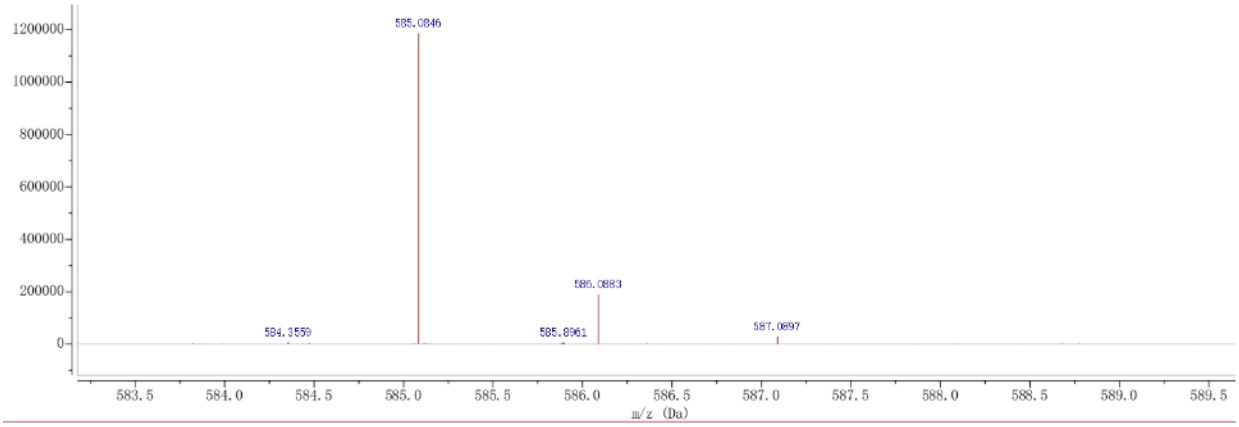

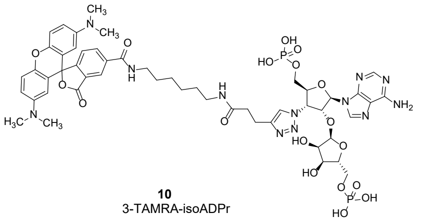

10
3-TAMRA-isoADPr

^{1}H-NMR (sample slightly contaminated with nBu4N^{+} (~0.3 eq.) during ion-exchange due to insufficient regeneration of resin)

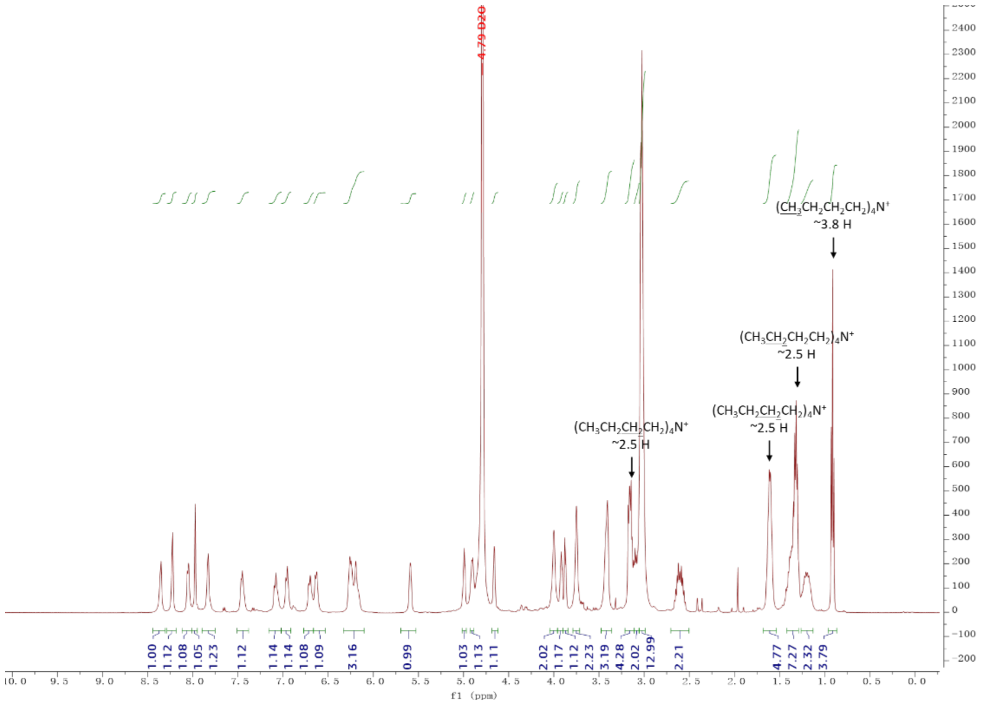

^{31}P-NMR

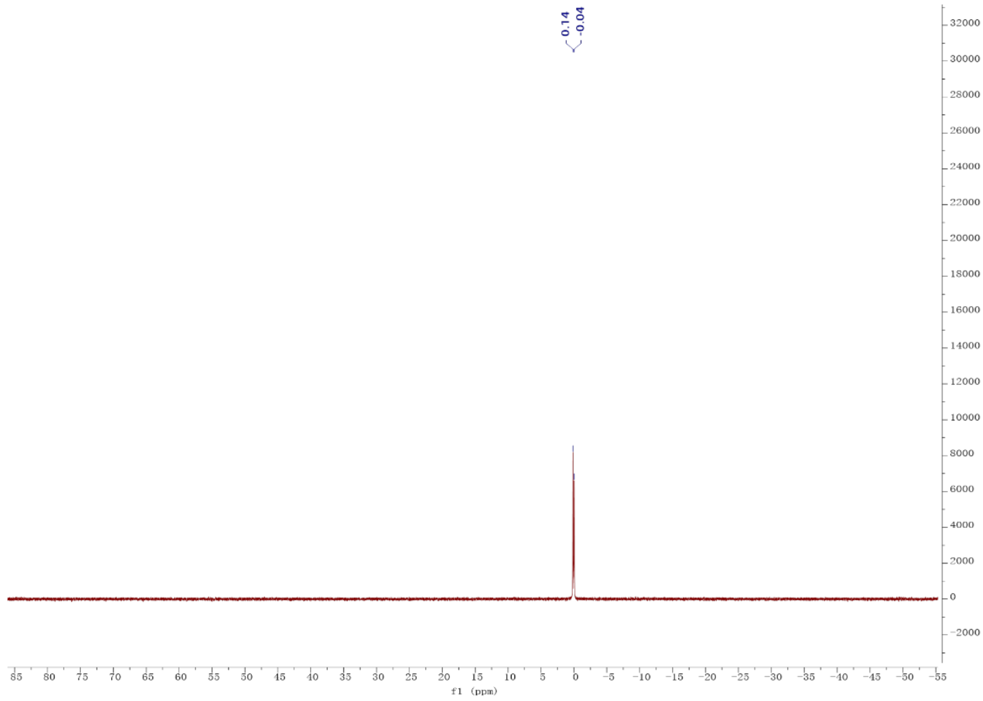

^{13}C-NMR

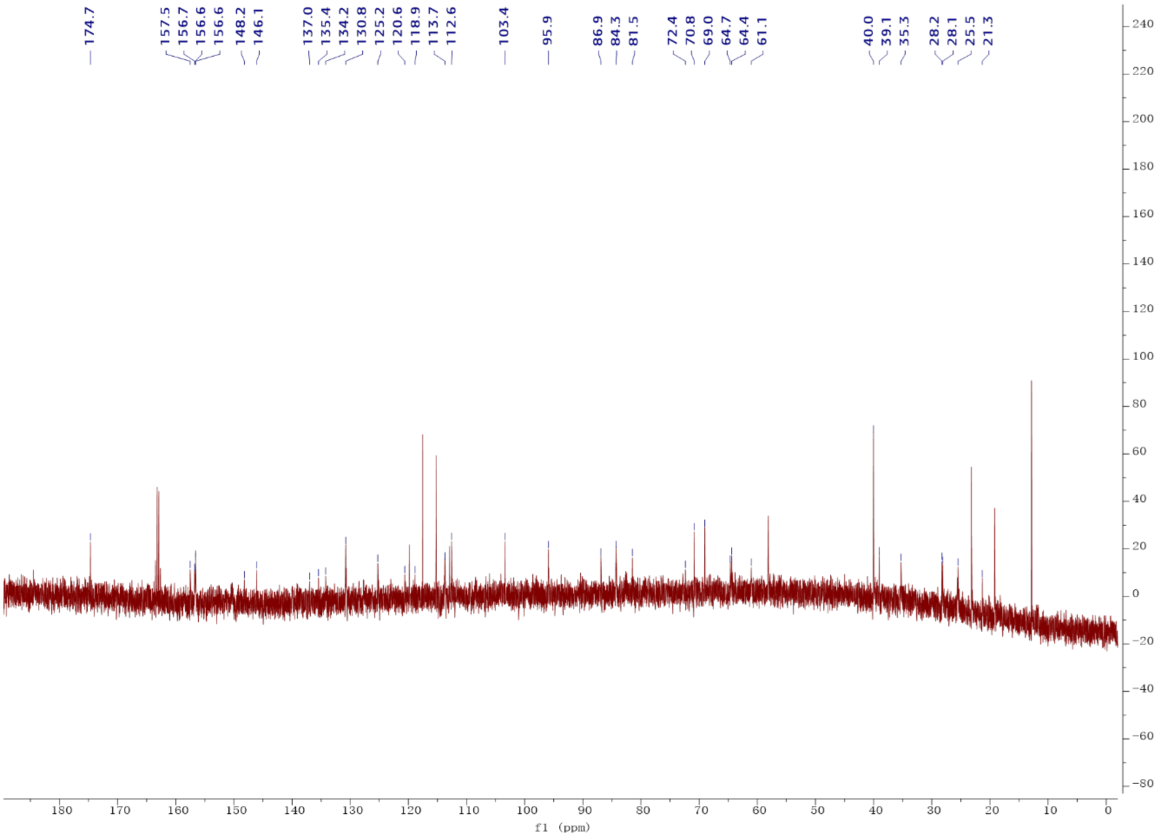
174.7
157.5
156.7
156.6
156.6
148.2
146.1
137.0
135.4
134.2
130.8
125.2
120.6
118.9
113.7
112.6
103.4
95.9
86.9
84.3
81.5
72.4
70.8
69.0
64.7
64.4
61.1
40.0
39.1
35.3
28.2
28.1
25.5
21.3
f1 (ppm)

COSY

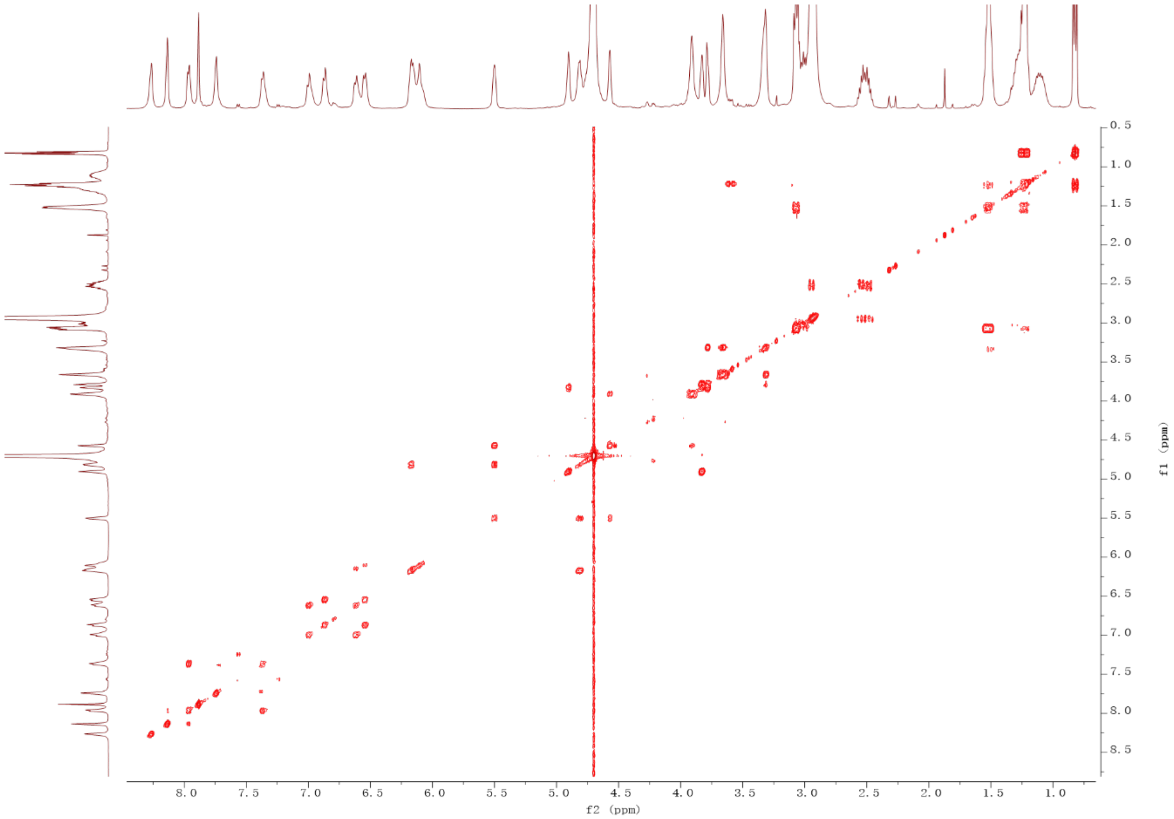
f2 (ppm)
f1 (ppm)

HSQC

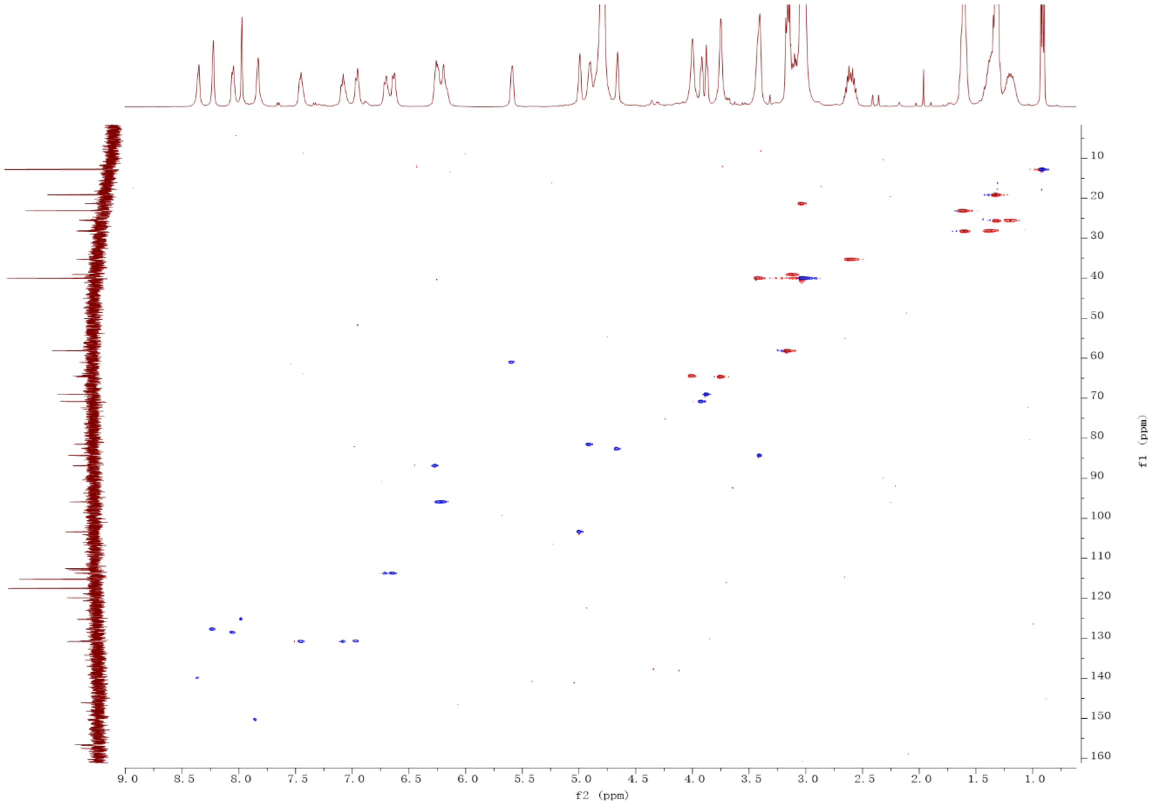

HRMS

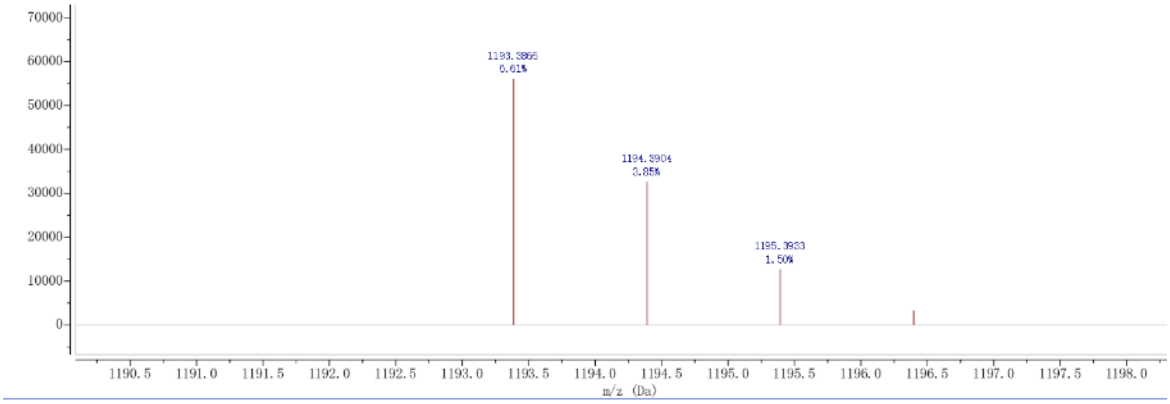

LC-MS

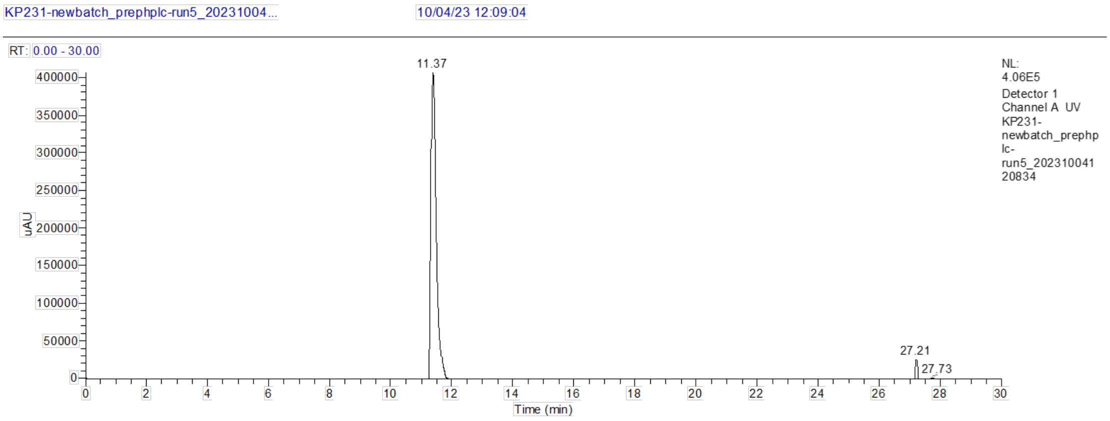

KP231-newbatch_prephplc-run5_20231004...
10/04/23 12:09:04
RT: 0.00 - 30.00
11.37
NL: 4.06E5
Detector 1
Channel A UV
KP231-newbatch_prephplc-run5_20231004120834
uAU
27.21
27.73
Time (min)

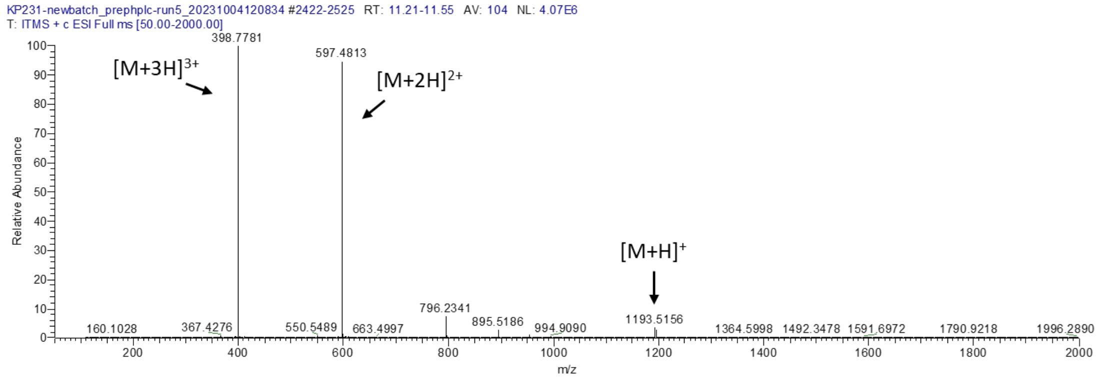

KP231-newbatch_prephplc-run5_20231004120834 #2422-2525 RT: 11.21-11.55 AV: 104 NL: 4.07E6
T: ITMS + c ESI Full ms [50.00-2000.00]
[M+3H]3+
398.7781
597.4813
[M+2H]2+
[M+H]+
160.1028
367.4276
550.5489
663.4997
796.2341
895.5186
994.9090
1193.5156
1364.5998
1492.3478
1591.6972
1790.9218
1996.2890
Relative Abundance
m/z

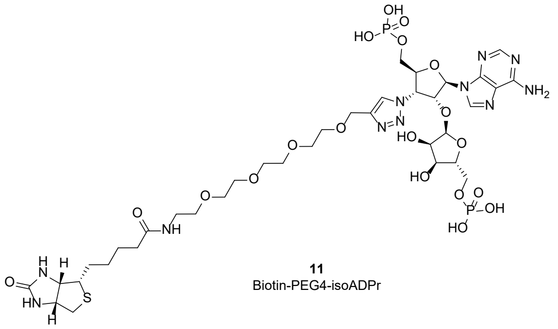

11
Biotin-PEG4-isoADPr

LC-MS

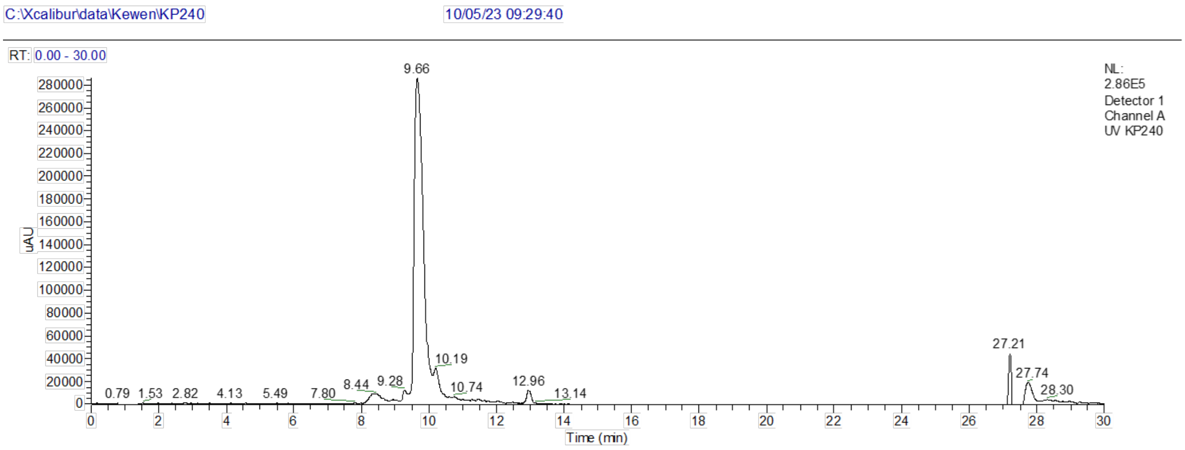

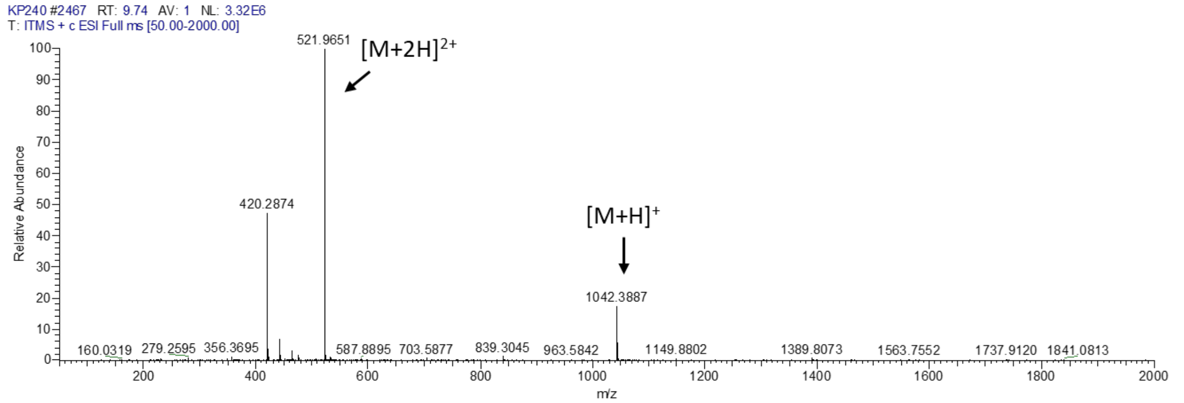

Supplement: Supplementary file 1 — cb3c00512_si_001.pdf [file cb3c00512_si_001.pdf]
